# Health and Economic Impacts of Eight Different Dietary Salt Reduction Interventions

**DOI:** 10.1371/journal.pone.0123915

**Published:** 2015-04-24

**Authors:** Nhung Nghiem, Tony Blakely, Linda J. Cobiac, Amber L. Pearson, Nick Wilson

**Affiliations:** 1 Department of Public Health, University of Otago, Wellington, Wellington South, New Zealand; 2 School of Population Health, University of Queensland, Brisbane, Australia; Erasmus University Rotterdam, NETHERLANDS

## Abstract

**Background:**

Given the high importance of dietary sodium (salt) as a global disease risk factor, our objective was to compare the impact of eight sodium reduction interventions, including feasible and more theoretical ones, to assist prioritisation.

**Methods:**

Epidemiological modelling and cost-utility analysis were performed using a Markov macro-simulation model. The setting was New Zealand (NZ) (2.3 million citizens, aged 35+ years) which has detailed individual-level administrative cost data.

**Results:**

Of the most feasible interventions, the largest health gains were from (in descending order): (i) mandatory 25% reduction in sodium levels in all processed foods; (ii) the package of interventions performed in the United Kingdom (UK); (iii) mandatory 25% reduction in sodium levels in bread, processed meats and sauces; (iv) media campaign (as per a previous UK one); (v) voluntary food labelling as currently used in NZ; (vi) dietary counselling as currently used in NZ. Even larger health gains came from the more theoretical options of a “sinking lid” on the amount of food salt released to the national market to achieve an average adult intake of 2300 mg sodium/day (211,000 QALYs gained, 95% uncertainty interval: 170,000 – 255,000), and from a salt tax. All the interventions produced net cost savings (except counselling – albeit still cost-effective). Cost savings were especially large with the sinking lid (NZ$ 1.1 billion, US$ 0.7 billion). Also the salt tax would raise revenue (up to NZ$ 452 million/year). Health gain per person was greater for Māori (indigenous population) men and women compared to non-Māori.

**Conclusions:**

This study substantially expands on the range of previously modelled salt reduction interventions and suggests that some of these might achieve major health gains and major cost savings (particularly the regulatory interventions). They could also reduce ethnic inequalities in health.

## Introduction

The risk factor of a “diet high in sodium” is one of the top two dietary risk factors for disease burden identified in the Global Burden of Disease Study 2010 [1]. Indeed, this sodium risk factor alone was ranked 11^th^ globally out of all risk factors considered (for a counterfactual of 1000 mg/day sodium intake). The scale of this problem has resulted in calls for “salt reduction” to be considered a public health priority, with it included in the top five priority actions for non-communicable disease (NCD) control internationally [2] and for reducing NCD inequalities [3]. Furthermore, in 2012 the World Health Organization (WHO) recommended a “reduction to <2 g/day sodium (5 g/day salt) in adults (*strong recommendation*)” [4].

Despite the above, the evidence relating to sodium and health is still perceived by some as controversial [5]. To some extent this relates to the uncertainty around the health benefits and risks of reducing sodium intakes below the 2300 mg level which is relatively low compared to current consumption (e.g., an Institute of Medicine Report [6]). But some commentators blame the perception of controversy on inadequate consideration of study limitations by authors, poor reporting by the media, and vested commercial interests exploiting the situation [7]. A recent example of a study casting doubt on aspects of the sodium and health relationship was published in 2014—the PURE Study [8]. But this observational study may have had limitations around the reliability of spot urine tests [9] and other issues [10], including reverse causation which could not be ruled out (as noted by the authors themselves). Indeed, what is more critical to consider is the totality of the evidence from published systematic reviews [11,12] and specifically: (i) the long-term trial data suggesting reduced sodium intake reduces cardiovascular (CVD) risk [13,14]; (ii) trial data showing this benefit for CVD mortality (albeit reducing sodium while raising potassium [15]; and (ii) data on reduced CVD risk in a nested observational study where participants had been randomised to a Mediterranean diet [16].

There is also a growing base of modelling studies which have considered the health gain and/or the economic aspects of dietary sodium reduction (see S1 file). Most of the published health economic evaluations indicate that sodium reduction interventions are likely to result in health gains while actually being *cost-saving* (e.g., by averting future health system costs). But there remains scope for methodological improvements in many of these studies, particularly around the need for more robust cost data and for better definitions around the interventions (Table A in S1 File).

Reducing health inequalities is also a common goal of health policy. Salt reduction has the potential to reduce inequalities in health, in absolute terms at least, due to the usually higher CVD rates among disadvantaged populations within countries—but this issue has not been well studied in modelling work to date (we identified just one study [17]). Also certain types of interventions to reduce sodium intakes have rarely been studied if at all. For example, there have been no studies on restricting the supply as per a cap-and-trade system on the food salt supply (as suggested elsewhere [18,19], and modelled with regard to sugar [20]), only one economic modelling study on a specific salt tax [21]; and rarely around labelling interventions [22]).

Given this picture, we aimed to advance the understanding of the effectiveness and cost-effectiveness of sodium reduction interventions by new modelling work. Our work benefited from a range of methods refinements and use of relatively good country-level disease data, which captures existing health inequalities by ethnicity (in a country, New Zealand, where these are prominent). We also used detailed individual-level health system cost data, which has recently become available for use in New Zealand. A total of eight different interventions were considered, which allowed comparisons between those used previously in New Zealand and the United Kingdom (UK), with more hypothetical ones.

## Methods

### Model structure and perspective

A model developed for studying CVD interventions in Australia [23], provided the base model. This model was built in Excel but, for our study as part of the BODE^3^ Programme of work (http://otago.ac.nz/bode3), we converted it to a Markov macro-simulation model in TreeAge Pro version 2013. Results identical to the Excel model were obtained in TreeAge, prior to populating the model with New Zealand data. The simulated population was a closed cohort of the New Zealand population aged 35 years and older (2.3 million people), modelled from the baseline year (2011) to death or age 100 years.

The Markov model has four primary health states, with annual transition rates capturing incidence and case-fatality for coronary heart disease (CHD) and stroke events (see the diagram in an online Technical Report [24] on the BODE^3^ website). Essentially, proportions of each age/sex/ethnicity cohort occupy the states of: being “healthy” (i.e., not having CVD), having a form of CVD (CHD or a type of stroke), or death, in each annual cycle.

In terms of modelling background disease trends we took the same approach as the New Zealand Burden of Disease Study (NZBDS) [25], and assumed a continued decline in incidence rates for both CHD and stroke of 2.0% annually, and also a 2.0% reduction in case-fatality annually (i.e., reflecting improved treatment and management). We extended this projection from 2016 (NZBDS end estimate) to the year 2026 and then held the incidence and case fatality rates constant.

Background population mortality was assumed to decline at a somewhat lower rate than for CVD with a 1.75% annual reduction for non-Māori, and 2.25% for the indigenous population of Māori (also out to the year 2026), then 0% per annum decline for both ethnic groupings thereafter. The justification for these trends is detailed in our Protocol [26].

A health system perspective was used. Costs and benefits beyond the health system (e.g., productivity gains from preventing premature deaths of workers) were considered out of scope as these are more relevant to a societal perspective. However, additional health system costs arising from extra life expectancy in the future attributable to the impact of the modelled interventions were included in the baseline analyses. Costs were calculated in 2011 New Zealand dollars and a 3% discount rate was applied to costs and future health gain (with the discount rate varied in scenario analyses: 0% and 6%). OECD 2011 purchasing power parties [27] were used for calculating results in US$ for international comparisons.

Our approach to cost-effectiveness analysis was that of the “generalised cost-effectiveness analysis” as developed for the WHO [28]. In this approach, all interventions (including current practice) are evaluated against a theoretical “do nothing” comparator (i.e., doing none of the interventions of interest in the analysis). This approach allows explicit estimation of the cost-effectiveness of current practice (if included as intervention), and so it avoids artificially making an intervention look more favourable if compared against inefficient current practice. Therefore, we back-calculated disease rates under the “do nothing” scenario using the same parameters of intervention effectiveness, adherence and costs that are used in the cost-effectiveness analyses (in this case for the Dietary Counselling and the Endorsement Label Programme interventions—as detailed below).

### Input parameters

Input parameters shown in Table 1 are summarised in the text below, and also explained in further detail in an online Technical Report.[24].

**Table 1:** Input parameters to the modelling: selected baseline and epidemiological parameters

Variable	Sources and key details	Key values and uncertainty
**Baseline variables in 2011**		
Sodium intake	Source: New Zealand (NZ) nutrition survey data [53], with significant variation by sex, but not by ethnicity or age (for adults). No trend under business-as-usual (BAU) specified, given no notable trend since the 1980s [54].	4013 mg/d for men and 3115 mg/d for women (nil uncertainty; rather uncertainty around the intervention associated reduction was considered—see below)
Incidence, prevalence and case-fatality data for CHD and stroke	Calculated using linked HealthTracker data, with coherency checks using DisModII and smoothing with regression as required. Future annual percentage change (APC) in incidence and CFR were both set at -2.0% each as per the NZBDS.	See online reports for details [24,30].
Morbidity (disability weights [DW])	From GBD2010 [32], with modification to NZ [25] and slight variation by age and ethnicity (see an online report [24] for details).	CHD = 0.081, Stroke = 0.226, (For uncertainty see: [24]).
Baseline health system costs for CHD and stroke states, and non-diseased states.	Calculated from HealthTracker data by sex and age in 2011 for people: (a) without either CHD or stroke; (b) with CHD only, and *excess* to (a); (c) with stroke only, and *excess* to (a). (See an online report [24] for details).	Examples for 60 year old females (gamma distribution with SD = 10% of mean): (a) NZ$2,381; (b) NZ$16,258 for the first year, NZ$5,395 for second and subsequent years; (c) NZ$20,553 and NZ$5,991 for stroke.
**Epidemiological associations**		
Change in systolic blood pressure (sBP) (in mm Hg) for each 100 mmol/d change in sodium intake	Derived from the regressions models developed by Law et al [33]. The small differences in BP by ethnic group did not justify separate modelling by ethnicity (higher in Māori by 3 mm Hg for systolic BP and 4 mm Hg for diastolic BP in both sexes compared to non-Māori [46]). Also of note is that no trend in BP into the future was considered given the unclear picture in NZ (of a downward trend in population BP levels from 1982 to 2002 and then an upward trend from then 2008/09) [46]. We also considered the results of another meta-analysis by He and Macgregor [11] in scenario analyses.	For men and women by age-group in sBP (mm Hg) change: 30–39: 5.5; 40–49: 6.6; 50–59: 9.2; 60–69: 10.3
Relationship between blood pressure and CVD risks	We used the results of a meta-analysis of 61 prospective studies by Lewington et al [34]. These results were considered to be more generalisable to the general population than those from a meta-analysis by Law et al 2009 of 147 RCTs of blood pressure-lowering drugs [35].	The hazard ratio for a 20 mm Hg reduction in systolic BP ranged from 0.49 to 0.67 for CHD and from 0.38 to 0.67 for stroke (depending on age). For uncertainty: SD = +/- 10% of the point estimate for each age group.

#### Incidence, prevalence and case-fatality

The estimated incidence, prevalence and case-fatality rates of CHD and stroke (ischaemic and haemorrhagic) were calculated across all combinations of sex, age-group (35–39, 40–44, … 95+ years) and ethnicity (Māori; and non-Māori). Data came from Ministry of Health data, called ‘HealthTracker’ [29], which is a collection of linked administrative datasets of publically-funded health system events. This includes hospitalisations, mortality, cancer registrations, mental health and addiction service use, pharmaceutical and laboratory claims, primary health care enrolment, and outpatient/emergency department visits for the entire New Zealand population with costs attached. But gaps in HealthTracker data exist in specific areas (e.g., some private sector expenditure and the health-related aspects of residential care) and so we scaled up both the CVD disease costs (CHD and stroke) and the annual health system costs for the non-diseased population. For the disease costs we scaled up HealthTracker costs across all age groups by 1.2. For the non-diseased population, costs were multiplied by 1.1, 1.2, 1.3 for the 65–74, 75–84 and 85+ age groups respectively to capture the estimated missing data of funding residential ‘disability support services’ care funded by the government (Vote:Health), but not yet captured in available data. All costs include the costs in the last six months of life.

Validation of model parameters and the final model outputs (relative to two official data sources) are detailed in an online Validation Report [30]. This additional work also involved parameter coherence checking, using the epidemiological software program DisMod II [31]. Of note, because of a 10 year look back period we could use in data analyses, our empiric estimates of prevalent disease are probably low (as we do not capture earlier incidence cases with no subsequent health event), and therefore our estimated case-fatality rates may be too high (as the ‘prevalent’ denominator is too low) when applied to our Markov model that projects out multiple decades. The DisMod checks possibly supported this concern for stroke. Therefore, we include reduced case-fatality rates as a scenario analysis (see below).

#### Morbidity and disability weights

Overall morbidity, by sex, age and ethnicity, was quantified in the model using the years of life lived with disability (YLDs) from the NZBDS [25], divided by the population count to give ‘prevalent’ YLDs. Disease-specific morbidity was assigned in each disease state (e.g., CHD and stroke), as the total comorbidity-adjusted YLDs for that disease divided by the prevalent population. The health status valuation used to calculate these YLDs were disability weights derived from the Global Burden of Disease study (GBD2010) using pair-wise comparisons from multi-country surveys [32], as opposed to, say, disutilities from the EuroQol. These disability weights are on a scale from 0 (full health) to 1.0 (death)—and included uncertainty (for details see the online Technical Report [24]). As per other BODE^3^ work we assumed no future underlying trend in morbidity burdens (i.e., both the size of the weights and the background level of non-CVD morbidity were assumed constant into the future). Of note is that the use of these weights limited the maximum QALYs that would be gained with increasing age. For example, an average Māori woman aged 60–64 has an expected level of disability of 0.288, meaning a year of life gained in this population group has a maximum value of 0.712. QALYs were cumulatively tallied for the life-span of the modelled cohort.

#### Intervention specification and parameters

We considered eight different interventions of which some were voluntary (e.g., dietary counselling, a labelling programme and a campaign run in the UK) and others were mandatory (requiring national laws for: legal limits on sodium in processed foods, a salt tax, and a sinking lid on the supply of salt to the New Zealand market). The details of these are in Table 2 and in Table B in S1 File.

In brief, for each of these interventions a reduction in sodium intake was linked to a reduction in systolic BP based on values derived from the regressions models developed by Law et al [33]. A reduction in systolic BP was then linked to a reduced probability of adverse health outcomes as per a meta-analysis of 61 prospective studies by Lewington et al [34] (i.e., though we also used the results of another meta-analysis in a scenario analysis [35]).

**Table 2:** Input parameters relating to the interventions effects (for further details see S1 File)

Intervention	Sources and extra details	Key values and uncertainty (average adult)^a^
**Counselling:** Dietary counselling by dietitians to reduce sodium intake (part of current practice).	The data obtained for the NZ setting are detailed in an online report [55]. For the effect size on sodium we used the results of the trials included in a 2013 Cochrane systematic review [56].	For the per hour impact of counselling: 7.6 mmol/d reduction (with uncertainty based on the initial trials in the Cochrane review. SD = 0.8 mmol/d). Normally distributed. Total amount of counselling in NZ: 4600 h/year (SD = 920). Gamma distribution.
**Endorsement Label Programme:** A programme involving an endorsement label (part of current practice).	The non-governmental organisation “the Heart Foundation” runs an endorsement label programme called the “Tick Programme”. Its estimated impact are in an online report [57] and published letter [58].	Effect size: 1.7 mmol/d reduction overall (38 mg/d) with SD at +/- 20% (-1.0 to -2.3 mmol/d). Normally distributed.
**Mandatory-3G:** Mandatory reduction of sodium in the manufacture of breads, processed meats and sauces	Based on the relative contributions of sodium to the NZ diet (based on national nutrition survey data) we estimated the impact of a hypothetical mandatory reduction of sodium in three groups of processed foods: breads, processed meats and sauces (i.e., the top three categories for sodium intake in NZ). A 25% reduction of sodium in each group was assumed to result from setting mandatory upper levels for sodium, giving a reduction in intake of 296 mg/d (12.9 mmol/d).	Effect size: 12.9 mmol/d reduction overall with SD at +/- 10% of this. Normally distributed.
**Mandatory-All:** Reduction of sodium in all processed foods by 25%.	As above for the Mandatory-3G intervention, except the 25% reduction was applied to all major types of processed foods (i.e., excluding sodium intakes from: fresh fruit and vegetables, fresh fish and meat, and also salt added in cooking and at the table). The estimate obtained was a reduction of sodium intake of 525 mg/d or 22.8 mmol/d (equivalent to 1.4 g/d out of 9.1 g/d salt intake currently or 15% of current adult intake).	Effect size: 22.8 mmol/d reduction overall with SD at +/- 10% of this. Normally distributed.
**UK Package:** The mix of media campaign, voluntary food reformulation and food labelling changes	The intervention was that actually used in the 2003–2009 period in the UK [59], but applied on a same per capita basis to NZ. This overall programme resulted in a 15% reduction in 24-hour urinary sodium over seven years in the adult population. We used this reduction in our modelling for the NZ population i.e., a 15% reduction in dietary sodium intake over seven years. In the baseline model we assumed that the benefit would stay in place for the lifetime of the modelled cohort (given the longer-term evidence from countries such as Finland [60]).	Effect size: 3.2 mmol/d reduction per adult annually over the seven year period (22.7 mmol/d overall) with SD at +/- 10% of this. Normally distributed.
**UK Mass Media Campaign:** Just the mass media campaign part of the UK Package	The mass media campaign component of the UK Package (as per directly above) was applied on the same per capita basis to NZ. There is evidence that this media campaign increased the proportion of UK adults who made an effort to cut down on salt (i.e., from 34% to 43%) and those trying to reduce salt by checking food labels also increased (i.e., from 29% to 50%) [59]. Overall, however, the media campaign has been described as being “not very effective in the long term” [59]. Given this information, and the other actions occurring at the time (industry food reformulation) we assumed a relatively modest role for the campaign—at around 30% of the total package effect size (range in scenario analyses of 15% to 45%). This range is very approximate but has been informed by expert opinion (Personal communication with He and Macgregor who have studied the UK campaign [59]).	Effect size: 0.97 mmol/d reduction per adult annually over the seven year period (6.8 mmol/d overall) with SD at +/- 30% of this. Normally distributed.
**Salt Tax:** An excise tax is applied and increased up to the point where the recommended level of sodium intake is achieved	We modelled a hypothetical intervention in which a law was passed requiring an excise tax on salt that would be applied in increasing amounts annually until a target level of population salt intake of 2300 mg/d (5.9 g salt/d) per adult was achieved (the level recommended for NZ adults [61]). We used a price elasticity (PE) for demand of salt from the literature of: -0.1 [62] (varied in scenario analyses). We set the tax levels so that the reduced demand in any one year would never exceed 20%. This meant that it took 10 years to reach the 2300 mg/d target. In the baseline model we assumed that the benefit would stay in place for the lifetime of the modelled cohort. Scenario analyses included a range of other options.	Effect size: Variable annual reductions to keep under the maximal level of 20% change in any year. The highest reduction was in the first year at 6.5 mmol/d per adult.
**Sinking Lid:** The amount of food-grade salt released onto the NZ market is reduced annually to the point where the recommended level of sodium intake is achieved	In this hypothetical intervention, a law was enacted requiring a stepwise reduction in the amount of food-grade salt released to the market (i.e., as released by NZ’s single salt manufacturer). The reduction continued until the target level of 2300 mg/d per adult was achieved (as per the Salt Tax). In the baseline model we assumed that it would take six years to achieve the target and that the benefit would stay in place for the lifetime of the modelled cohort. Scenario analyses included a range of other options.	Effect size: A reduction in sodium consumption of 9.0 mmol/d per adult each year (until the target is reached).

^a^ Values given for the average adult. In the modelling we adjusted these values for men and women by ratios of 4013/3544 and 3115/3544 respectively, given the variation in sodium intakes (in mg) according to the nutrition survey data [53].

#### Costing of intervention scenarios and health system costs

We considered the net cost, which is the intervention costs plus health system costs throughout the lifespan of the modelled cohort (i.e., we captured additional health costs associated with any extra lifespan generated by the interventions). Specific details for the costing of the interventions are provided in Table 3. For health system costs, the ‘business as usual’ ones were determined by strata of sex and age using HealthTracker data, which links cost estimates to all health events. From this dataset it was possible to calculate the 2011 costs for the first year of CHD and stroke, and then the average annual cost for the second and subsequent years (S2 file). Furthermore, given that CVD is a relatively important part of baseline health system costs, we adjusted the baseline health system costs experienced by the “healthy” component of the modelled population, to remove the CVD-attributable cost component (to avoid double-counting).

**Table 3:** Input parameters relating to the interventions costs.

Intervention	Sources and comments	Key values and uncertainty (average adult)
**Counselling**	We considered dietitian delivered counselling (private sector and DHB funded). As detailed in an online report [55] this was estimated at NZ$575,000 per year (for 2011). A key parameter was the DHB-funded dietitian time (as per official national DHB funding values) was NZ$115.89 per one hour consultation (assumed SD = 10% = NZ$11.59). The annual costs continued for the lifetime of the cohort (to the year 2076).	Cost: NZ$575,000 per year, equivalent to NZ$0.24 per adult in NZ
**Endorsement Label Programme**	For the Programme costs, we treated the programme as part of New Zealand health sector activity. We used the annual operating costs reported by the Heart Foundation of NZ$621,000 for the calendar year 2011 (see an online report [57]). These cover the running of the Programme and product testing (with the food industry making payments to the Heart Foundation for participation in the Programme). The annual costs continued for the lifetime of the cohort (to the year 2076).	Cost: NZ$621,000 per/y with SD at +/-10% of the estimate (i.e., SD = 62,100). Gamma distribution.
**Both “Mandatory” interventions**	The cost of enacting a new law was used, based on the average cost of new act in NZ [63] (with NZ$NZ dollar values reported separately [64]). That is, the cost of a new law was estimated NZ$ 3,680,000 (in 2011 dollars). In scenario analyses we assumed that the laws for these two interventions would have a limited life (e.g., a sunset clause at 20 years) at which time we assumed that sodium levels in foods would revert to their pre-intervention levels. In the baseline model we assumed no significant changes to current evaluation efforts by the NZ Government (nutrition surveys and food surveys) and negligible legal costs associated with non-compliance. Nevertheless, we performed a scenario analysis based on Australian estimates that covered both legislative changes and on-going enforcement costs. That is we used the NZ cost of a law plus added in half the cost of the Australian value (which covered by legislation and enforcement). That is half of AUS$ 0.49 per person per year (gamma distribution, SE = NZ$0.05). The Australian value is for the year 2008 (see supplementary information in Cobiac et al [23]) was derived from resource use estimates [65] and WHO unit costs (www.who.int/choice/costs/en/).	Cost: NZ$3,680,000 in 2011. Gamma distribution with SD of +/-25% of the point-estimate.
**UK Package**	The total cost of the mass media campaign that was run between 2003 and 2009 was UKP 20,043,445 [66]). This was equivalent to a cost of UKP 0.42/adult (for the UK in 2008) and we used this to calculate the cost per NZ adult in 2011. In addition, we used the per capita extra cost of a non-governmental group (“CASH”) which helped promote the UK Package of interventions and facilitate various accompanying publicity, and which had approximate expenditure from 1996 to 2011 of around UKP one million [59]. In a scenario analysis we considered that all intervention costs were 50% greater.	Total cost (NZ$): 12,100,000 in 2011. Gamma distribution with SD of +/-10% of the point-estimate.
**UK Mass Media Campaign**	As detailed above for the mass media component of the UK Package, and with a scenario analysis that considered that these intervention costs were 50% greater.	Total cost (NZ$): NZ$10,400,000 in 2011. Gamma distribution with SD of +/-10% of the point-estimate.
**Salt Tax**	As for the Mandatory interventions around sodium levels in food (as per above), we used the average cost of a new act in NZ (to introduce the excise tax on salt). The enforcement and compliance cost was not considered in our analysis given the single main producer of salt in NZ. If there were compliance issues—then this could be addressed via fines that were set at levels that typically covered enforcement and legal costs.	Cost: 3,680,000 in 2011. Gamma distribution with SD of +/-25% of the point-estimate.
**Sinking Lid**	As above for the Salt Tax (i.e., the cost of a new law to require the reduction in supply to the market).	As above.

As further context, New Zealand is a fairly typical OECD country in terms of health spending (at 10.0% of GDP—slightly more than the OECD average of 9.3%) [36]. But 83% of health spending was funded by public sources in 2011 (which is well above the average of 72% in OECD countries). Residential care for the elderly in New Zealand is largely funded from social welfare budget (and so is excluded from our analysis—given the health system perspective). Nevertheless, the residential care costs that relate specifically to health (i.e., residential care hospital facilities) is captured in our analysis (via scaling up from HealthTracker costs—see above).

Future trends in health costs were not modelled as these are considered very uncertain due to reasons around the New Zealand economy’s dependency on commodity prices, recent expansion in the role of the government’s pharmaceutical purchasing agency, and potential future trade agreements that might limit the government’s capacity to constrain health costs.

### Scenario and uncertainty analyses

We reran models (usually for expected values only) for a wide range of scenarios to assess the impact of components of the interventions and other structural assumptions (e.g., the discount rate). We also varied the background case-fatality rates for CVD (see above) and undertook a range of one-way uncertainty analyses and derived Tornado plots, using the 2.5^th^ and 97.5^th^ percentile values of input parameters. This was to assess which input parameter uncertainty contributed the most to uncertainty in the model outputs i.e., QALYs, net cost and incremental cost-effectiveness ratio (ICER).

A scenario analysis relating to equity considerations involved using for the Māori population, the lower background morbidity and mortality of the non-Māori population. This approach meant that Māori are not considered to be “penalised” in terms of the scope for future health gain due to poorer background health status relative to the non-Māori population. Further justification of such an approach has been detailed previously [37].

#### Interpretation of cost-effectiveness

There is no universally accepted threshold in the New Zealand setting for describing an ICER as being “cost-effective” or not. So we relied on WHO recommendations relating to GDP per capita [38], and used a nominal GDP per capita of NZ$45,000 in 2011 (US$29,600).

## Results

**Fig 1:**
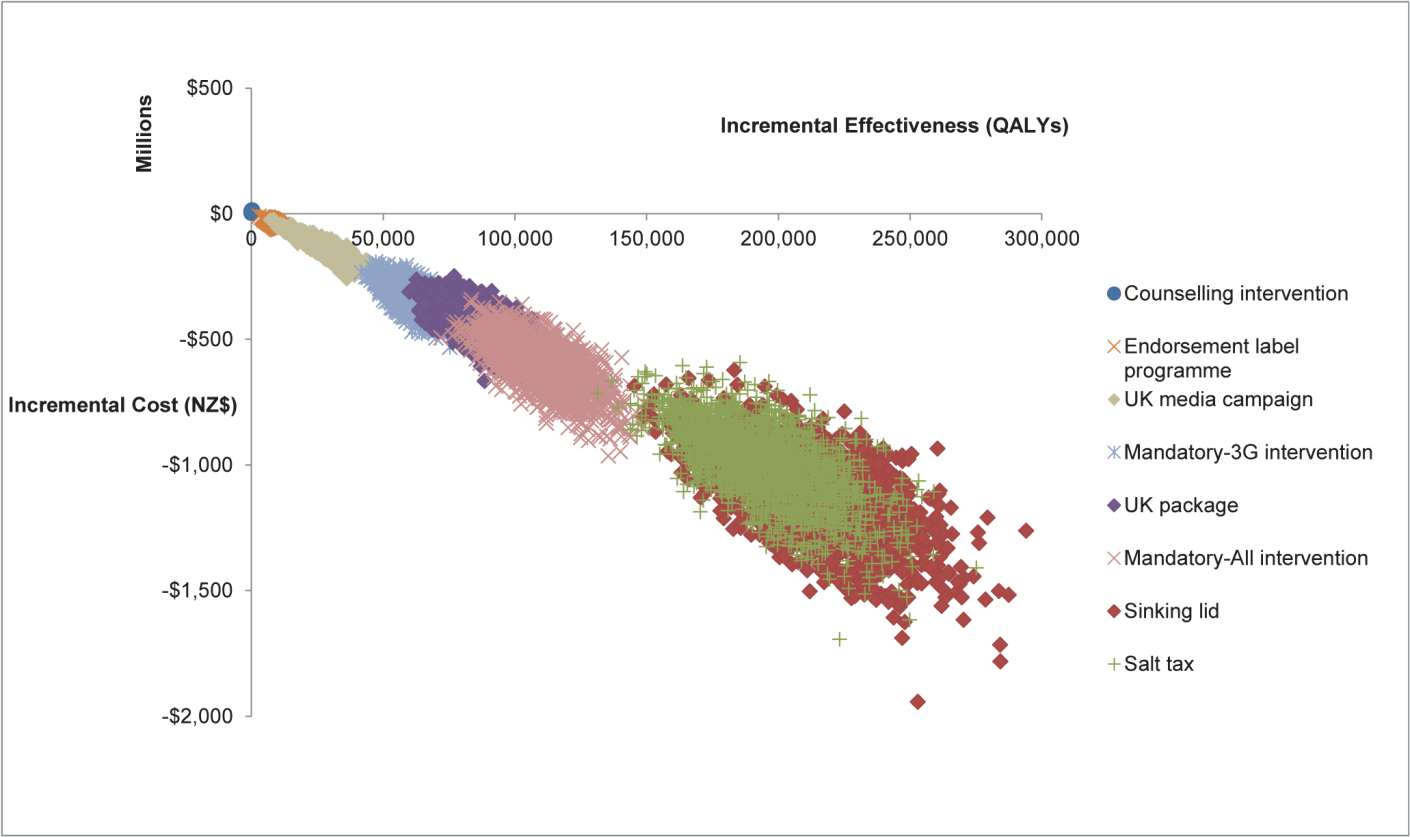
Cost-effectiveness plane with the eight salt-reduction interventions for the New Zealand adult population.

**Fig 2:**
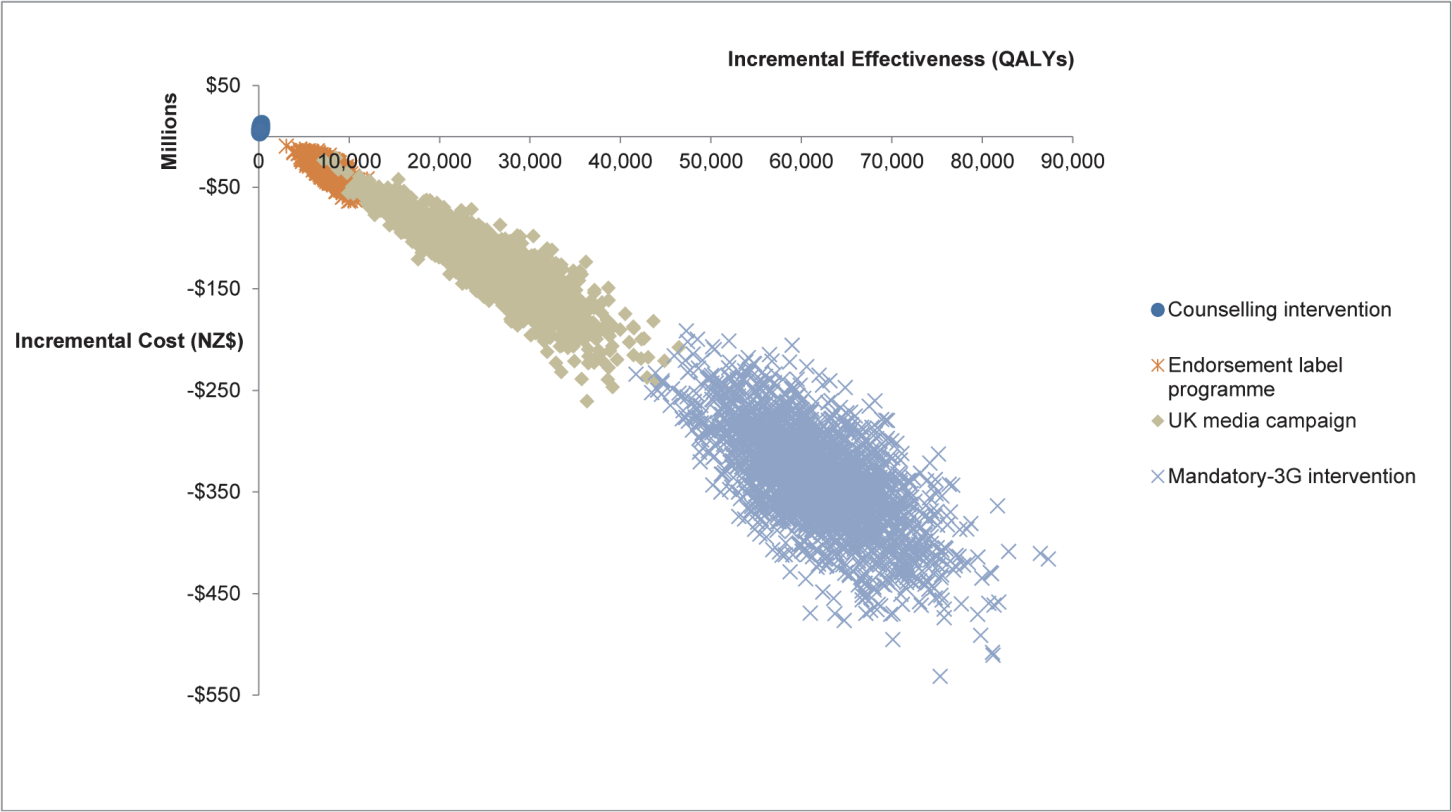
Cost-effectiveness plane with further detail on four of the salt-reduction interventions on the New Zealand adult population (for comparisons with the other interventions—see Fig 1).

The largest health gain was from the potential intervention of a Sinking Lid in food salt released to the market to achieve an average adult intake of 2300 mg sodium/day (Figs 1 and 2 and Table 4). It achieved 211,000 QALYs gained (95% uncertainty interval [UI]: 170,000–255,000). This QALY benefit was followed in descending order by that from a: (i) Salt Tax (195,000 QALYs gained); (ii) mandatory 25% reduction of sodium levels in processed food (“Mandatory-All”), (110,000); (iii) the package of interventions performed in the UK (85,100); (iv) mandatory 25% reduction in sodium levels in bread, processed meats and sauces (“Mandatory-3G”), (61,700); (v) Media Campaign as per the UK one (25,200); (vi) the voluntary Endorsement Label Programme as currently used in New Zealand (7900); and (vii) Dietary Counselling as currently used in New Zealand (200 QALYs gained).

**Table 4:** Population level results for the cost, health gain and cost-effectiveness of the interventions (95% uncertainty intervals in parentheses)^a^.

	Health system cost (NZ$; millions) for remainder of the cohort’s life	QALYs for remainder of the cohort’s life	Incremental cost-effectiveness ratio (ICER) (cost per QALY)
“Do nothing” comparator^b^	162,000 (145,000 to 181,000)	33,200,000 (33,000,000 to 33,400,000)	Not applicable
***Incremental to “Do Nothing”***			
Counselling	6.90 (4.20 to 10.20)	200 (100 to 330)	NZ$36,900 (22,400 to 62,500)
Endorsement Label Programme	-34 (-52 to -19)	7900 (5500 to 10,400)	Dominant
Mandatory-3G	-340 (-440 to -240)	61,700 (49,700 to 74,900)	Dominant
Mandatory-All	-600 (-800 to -440)	110,000 (87,500 to 135,000)	Dominant
UK Package	-440 (-570 to -320)	85,100 (69,600 to 102,000)	Dominant
UK Mass Media Campaign	-120 (-200 to -62)	25,200 (14,200 to 36,700)	Dominant
Salt Tax	-1000 (-1320 to -740)	195,000 (159,000 to 237,000)	Dominant
Sinking Lid	-1110 (-1460 to -830)	211,000 (170,000 to 255,000)	Dominant

^a^ Based on 2000 Monte Carlo simulations for the NZ adult population aged 35+ years and alive in 2011 modelled out to death or age 100. Numbers are rounded to two or three meaningful digits.

^b^ No intervention costs are included in this “do nothing comparator” (the costs of the currently existing programmes of “Counselling” and the “Endorsement Label Programme” are removed and are shown instead in the subsequent rows for results that are “incremental to ‘do nothing’”).

The Sinking Lid produced the highest discounted net savings of NZ$ 1.1 billion (US$ 0.7 billion) over the lifetime of the population. However, the Salt Tax was both cost-saving (NZ$ 1.0 billion) and would also actually raise NZ$ 452 million in revenue per annum by 2021. The only intervention not found to be cost-saving was Dietary Counselling. Nevertheless, it was still typically cost-effective with a mean ICER of NZ$ 36,900 (US$ 22,300) per QALY gained (95% UI: NZ$ 22,400–62,500).

Table 5 shows the overall cost results were largely driven by averted disease treatment costs for CVD, followed by the increased health system costs from extra life lived (as a result of the interventions).

**Table 5:** Types of costs (NZ$) by intervention (expressed per adult in 2011).

Intervention	Direct intervention cost	CVD health system costs	Non-CVD health system costs	Net cost
***Baseline***				
“Do nothing” comparator	–	16,000	54,500	70,500
Counselling	3.60	16,000	54,500	70,500
Endorsement Label Programme	4.10	16,000	54,500	70,500
Mandatory-3G	1.40	15,700	54,600	70,300
Mandatory-All	1.40	15,500	54,800	70,200
UK Package	4.70	15,600	54,700	70,300
UK Mass Media Campaign	4.10	15,800	54,600	70,400
Salt Tax	1.40	15,100	55,000	70,000
Sinking Lid	1.40	15,000	55,000	70,000
***Incremental to “Do Nothing” costs***				
Counselling	3.60	-1.00	0.40	3.00
Endorsement Label Programme	4.10	-36.5	17.5	-14.9
Mandatory-3G	1.40	-286	138	-147
Mandatory-All	1.40	-509	245	-263
UK Package	4.70	-385	191	-190
UK Mass Media Campaign	4.10	-115	56.6	-54.1
Salt Tax	1.40	-876	440	-435
Sinking Lid	1.40	-956	474	-481

### Heterogeneity by socio-demographics

The QALYs gained were higher and the cost-savings greater for younger age groups (<65 years) for all interventions except for Counselling (Table C in S1 File). The same pattern existed for the greater health benefit and greater cost-saving for men compared to women. In contrast to the other population groups, Counselling was not cost-effective for older ages (65+ years) and for women (Table C in S1 File). For all the interventions there was greater health benefit for Māori compared to non-Māori (e.g., 1.3 times more QALYs gained for the Mandatory-All intervention) (Table C in S1 File).

To facilitate a more detailed ethnic inequalities analysis, we present both model-estimated CVD mortality rates in 2021 (i.e., once the interventions have been operational for 10 years and have had a chance for their impact to play out and are fairly stable) and QALYs gained per person, by strata of sex, age and ethnicity in Table 6. This analysis used both the Counselling intervention and one of the more plausible of the hypothetical interventions: the Mandatory-All intervention. As per Table 6, the Counselling intervention had a negligible impact. The Mandatory-All intervention reduced CVD mortality rates more in absolute terms among Māori, but less in relative terms, resulting in estimated decreases in rate differences between Māori and non-Māori—but increases in the rate ratio. For example, among 50–54 year old men the Māori:non-Māori mortality rate difference decreased from 122 to 115 per 100,000 with the Mandatory-All intervention—but the rate ratio increased slightly from 4.46 to 4.48. Similar patterns were evident for women and older age groups.

**Table 6:** Ethnic inequality impacts after 10 years from two sodium reduction interventions (CVD mortality rates, rate ratios and rate differences, and QALYs gained for individuals given model structure assumptions and parameter inputs for selected age-groups).

			Do nothing				Counselling intervention				Mandatory-All intervention			
Sex	Age-group in 2011	Ethnic group	CVD mort-ality rate^a^	Rate diff^a^	Rate ratio^a^	QALYs (remaining life)	CVD mort-ality rate^a^	Rate difference^a^	Rate ratio^a^	QALYs gained per individual^b^	CVD mortality rate^a^	Rate difference^a^	Rate ratio^a^	QALYs gained per individual^b^
Men	50-54yrs	Non-Māori	35	0	1.00	16.0	35	0	1.00	0.000135	33	0	1.00	0.065
		Māori	157	122	4.46	12.7	157	122	4.46	0.000199	148	115	4.48	0.086
		Māori equity^**c**^	-	-	-	14.8	-	-	-	0.000248	-	-	-	0.109
	75-79yrs	Non-Māori	514	0	1.00	5.9	513	0	1.00	0.000027	502	0	1.00	0.036
		Māori	1235	721	2.40	4.5	1235	721	2.40	0.000028	1209	707	2.41	0.040
		Māori equity^**c**^	-	-	-	5.5	-	-	-	0.000037	-	-	-	0.051
Wom-en	50-54yrs	Non-Māori	21	0	1.00	16.7	21	0	1.00	0.000085	19	0	1.00	0.044
		Māori	98	77	4.75	13.6	98	77	4.75	0.000124	92	73	4.78	0.066
		Māori equity^**c**^	-	-	-	15.7	-	-	-	0.000156	-	-	-	0.074
	75-79yrs	Non-Māori	453	0	1.00	6.6	453	0	1.00	0.000026	439	0	1.00	0.032
		Māori	1077	625	2.38	5.1	1077	624	2.38	0.000025	1045	607	2.38	0.033
		Māori equity^**c**^	-	-	-	6.1	-	-	-	0.000034	-	-	-	0.043

^**a**^ CVD mortality rates in 2021, and rate differences in CVD mortality rates, per 100,000 population. The CVD mortality rates were calculated by dividing all CVD deaths generated by the Markov model in the year 2021 by the number of people who were alive in that year (because we expected that the mortality rates would be stable after 10 years of starting the interventions). Rate differences and ratios for Māori compared with non-Māori (within sex by age-group). All rates started as per those in 2011 (they decrease by 2% per annum up to 2031, then remain constant, in the actual Markov model).

^**b**^ QALYs gained are per individual in the relevant age/sex/ethnic group accumulated over the 10 year period from 2011 to 2021 (all discounted at 3%). These are over and above the total expected QALYs in remaining life in the “Do Nothing” scenario (also discounted at 3%).

^**c**^ In an “equity analysis” we applied non-Māori mortality rates and non-Māori levels of morbidity (prevalent years lived with disability [pYLDs]) to both Māori and non-Māori (this effectively expanded the envelope for potential health gain for Māori).

Considering the QALYs gained, there appear to be larger absolute gains for Māori at younger ages—consistent with the larger absolute reduction in CVD rates. However, for older 75–79 year olds there was little difference in QALYs gains between Māori and non-Māori, due to the higher background (competing) mortality and morbidity among Māori, *limiting* potential health gains from a CVD-only intervention. In the additional “equity analysis” that we performed (i.e., to avoid “penalisation” of Māori we applied the lower non-Māori mortality and morbidity rates to Māori—see *Methods*), QALYs gained by Māori were substantially greater than for non-Māori.

### Scenario analyses around the interventions

For some of the scenario analyses considered, the Counselling intervention was not always cost-effective (e.g., at the 6% discount rate) (Table D in S1 File and Table E in S1 File). This was also the case when we used the results of another meta-analysis for the relationship between sodium intake and blood pressure [11] (i.e., this pushed up the ICER to NZ$ 64,800 per QALY gained). Nevertheless, for all other seven interventions these remained cost-saving in the scenario analyses considered (including adjustments to the case-fatality rates for CVD). Further comments on these analyses are in the S1 file.

### Uncertainty analyses

Tornado plots show how input parameters had an impact on the model’s incremental costs, QALYs gained, and ICERs for the Counselling and Mandatory-All interventions (Fig A in S1 File). For both interventions, uncertainty for QALYs and costs was particularly driven by the uncertainty in the level of sodium reduction from the intervention (i.e., those parameters described in Tables 2 and 3), followed by the uncertainty in the relative risk for the BP-stroke association. The impact of uncertainty in the relative risks associating stroke with BP was much greater than the parallel impact for CHD due to health gains being mediated more by stroke than CHD.

## Discussion

### Main findings and interpretation

This study adds to the existing literature to provide additional modelling-level evidence that legislation-based interventions for reducing sodium in the food supply would provide large health gains and also large cost-savings for a health system. The relatively greater health benefit from mandatory (vs voluntary) interventions is consistent with previous modelling work (e.g., [39,40]) and is not surprising given the strong scientific basis for the effectiveness of public health laws in general [41,42], and simply because interventions that change the food environment tend to have large reach and don’t involve individual-level behaviour changes and the costs of health professional time (e.g., as required for counselling interventions).

The net cost savings achieved for most interventions are consistent with much of the previous modelling literature around salt interventions and can be attributed to the relatively low cost of passing legislation (especially in the New Zealand setting), the cost savings from preventing CVD disease in the short and medium-term, and the fact that discounting partly erodes the impact of the more temporally distant extra health costs from increased lifespan.

Given the quality of New Zealand data by ethnicity, we were able to examine likely ethnic inequality impacts of these interventions. Assuming that the effect sizes (i.e., association of BP with stroke/CHD, association of changing salt intake with BP, and association of intervention with salt reduction) are similar across ethnic groups, but allowing for the higher age-specific CVD incidence rates among Māori, our modelling results suggest that CVD mortality rate differences will decrease in the future with a mandatory salt reduction strategy and that QALY gains will be greater for Māori. That is, a mandatory salt reduction intervention appears to be an inequality reducing intervention in absolute terms, consistent with research elsewhere on population-wide interventions on CVD risk factors [43,44]. However, the future CVD mortality rate ratios may increase; it is not uncommon for absolute difference to decrease, but relative differences to increase over time, with respect to inequalities [45]. This inequality reducing benefit of sodium reduction interventions appears to have only been detailed once before in modelling work—for African American men and women in the US [17]. Furthermore, our modelling of the inequality reduction effects might actually underestimate the benefit for Māori since we did not consider the slightly higher baseline BP for Māori (albeit only 3 mm Hg systolic BP [46]).

The results in this study probably have a reasonable level of applicability to other countries—given that high sodium intakes are a risk to health in virtually every country and most governments have the potential powers to legislate around sodium in the food supply. But for some of the interventions there will of course be specific considerations around feasibility and effectiveness (see S1 file). Furthermore, there can be tremendous variations in the net health system cost depending on how diseases are costed, model structure assumptions, and how the timing of costs is dealt with (see van Baal et al for an analysis of many of these aspects [47]). For example, the full inclusion of residential care costs as per analyses for the Netherlands may contribute to making some preventive interventions relating to tobacco control [48], and obesity control [49], less likely to be net cost saving.

### Study strengths and limitations

This study included various improvements compared to previous disease and health economic models around sodium reduction (particularly in terms of cost data, but also in terms of considering such issues as ethnicity—see *Introduction*). Some of the interventions had not been subjected to health economic modelling before (e.g., the Sinking Lid) or else only modelled rarely (e.g., a media campaign to lower sodium, the Endorsement Label Programme [22] and the Salt Tax [21]). Although NICE reported that the UK interventions around sodium would be cost-saving [50], no economic modelling details have been published on the UK Package.

Nevertheless, as per other such modelling work there are many limitations. These are expanded on in the S1 file, but to summarise they include limitations around: (i) model structure and indeed our uncertainty estimates do not capture uncertainty arising from “model structure uncertainty”; (ii) limitations around input parameters (e.g., particularly relating to limitations with current HealthTracker costs and some epidemiological data (e.g. prevalence of CVD), and for the more theoretical interventions such as the Sinking Lid); (iii) unknowns in public and industry responses (e.g., compensatory behaviours in response to perceived reduced saltiness of processed foods); and (iv) just taking a health system perspective (e.g., ignoring the economic benefits of preventing premature deaths in workers).

### Potential research and policy implications

Given the limitations with such modelling work as this, additional research is clearly desirable, particularly around an expanded set of plausible interventions and for using additional real-world data on the impact of down-regulating sodium in processed foods (e.g., as per recent laws in South Africa [51] and various European countries [52]). Other potential interventions that could be modelled further (for health gain and cost-effectiveness) include more general “junk food taxes”, and/or subsidising fruit and vegetables.

Nevertheless, waiting for such additional research is not critical if policy-makers are seeking to address the NCD epidemic in their countries and to achieve large financial savings. An optimal strategy might be to introduce a salt tax and then to use the tax revenue gained for additional health-promoting interventions (e.g., subsidising fruit and vegetables or providing healthier school lunches for children). But some policy-makers might be more interested in using the Sinking Lid approach, and this could be argued for on the grounds of the growing international experience with administering cap-and-trade systems for greenhouse gases and other air pollutants (such as sulphur and nitrogen oxides in the USA).

Other policy-makers may wish to start by regulating just the top sources of sodium by food category (as per the Mandatory-3G intervention). This would probably be easier to implement and evaluate than the more comprehensive, but possibly fairer, Mandatory-All approach.

## Conclusions

In modelling work that had a range of improvements on previous models (particularly in terms of cost data) it was found that the use of mandatory controls on sodium in the food supply delivered both major health gains and major cost savings. Absolute health gain per person was greater for Māori men and women compared to non-Māori. Therefore such interventions could also reduce ethnic inequalities in health.

## Supporting Information

S1 File Supporting Information—Main file. (DOCX)

S2 File Supporting Information—Costs for the salt model. (DOCX)
