# Supplementary material for: Health and Economic Impacts of Eight Different Dietary Salt Reduction Interventions
Source: PLoS One. 2015 Apr 24;10(4):e0123915. doi: 10.1371/journal.pone.0123915 (PMC4409110; doi:10.1371/journal.pone.0123915)
Supplement: S1 File — (DOCX) [file pone.0123915.s001.docx]

**Supporting Information – Main File (S1)**

**(For Nghiem et al – “Health and Economic Impacts of Eight Different Dietary Salt Reduction Interventions”)**

Contents

[Previous modelling studies 1](#_Toc415556403)

[Additional methods details 4](#_Toc415556404)

[Additional Results 7](#_Toc415556405)

[Scenario analyses – extra methods and results 9](#_Toc415556406)

[Uncertainty analysis – tornado plots 14](#_Toc415556407)

[Further details on the limitations of this modelling work 17](#_Toc415556408)

# Previous modelling studies

**There have been quite a number of modelling studies which have considered both the health gain and economic aspects of dietary sodium reduction [**[**1-7**](#_ENREF_1)**]. M**ost of the published health economic evaluations indicate that sodium reduction interventions are likely to result in health gains while actually being *cost-saving*. Such studies have been conducted for: Argentina [[8](#_ENREF_8),[9](#_ENREF_9)], Australia [[10](#_ENREF_10),[11](#_ENREF_11)], England [[12](#_ENREF_12)], England and Wales [[13](#_ENREF_13)], the UK **[**[**14**](#_ENREF_14)**],** Norway [[15](#_ENREF_15)], the US **[**[**16**](#_ENREF_16)**,**[**17**](#_ENREF_17)**]**, and for four Eastern Mediterranean countries [[18](#_ENREF_18)]. This cost-saving aspect arises because of averted future health system costs, but is also sensitive to what costs (and discount rates) are included in the study. **For studies not reporting cost-saving results, the pattern is suggestive of sodium reduction interventions being very cost-effective. That is, estimates include:** US$118 per disability-adjusted life-year (DALY) averted **from a health education campaign in Vietnam [**[**19**](#_ENREF_19)**]; US$23 per DALY** averted from legislation to decrease sodium content in processed foods and appropriate labelling in selected western-European countries [[20](#_ENREF_20)]; and I$293 per DALY averted from sodium regulation in food in Mexico [[21](#_ENREF_21)].

But there remains scope for methodological improvements in many of these studies, particularly around the need for more robust cost data and for better definitions around the interventions (as two previous reviews [[22](#_ENREF_22),[23](#_ENREF_23)] and our own examination of recent studies detailed in Table A).

Table A. Health economic analyses of salt reduction interventions reported for developed countries (cost-benefit, cost-effectiveness or cost-utility analyses published from January 2010 to January 2014, and compared to this NZ study)^a^

| **Characteristic** | **This NZ study** | **Australian studies** | **England** | **England & Wales** | **Finland** | **US** | **US** |
| --- | --- | --- | --- | --- | --- | --- | --- |
| **Reference** | This article | Cobiac et al [[10](#_ENREF_10),[11](#_ENREF_11)] | Dodhia et al 2012 [[12](#_ENREF_12)] | Barton et al 2011 [[13](#_ENREF_13)] | Martikainen et al 2011 **[**[**6**](#_ENREF_6)**]** | Smith-Spangler et al 2010 [[17](#_ENREF_17)] | Bibbins-Domingo et al 2010 **[**[**16**](#_ENREF_16)**]** |
| **Perspective** | Health sector | Health sector | “Health service” | Health sector | Societal (included productivity losses) | Societal (but largely health – no productivity loss) | Health sector |
| **Population / time horizon** | To death or age 100 years of cohort (35y+) | To death of cohort aged 35+ | 10 years for cohort aged 16+ | 10 years for cohort aged 40-90y | For the years 2010-2030 (to death or age 75 years in those aged 30-74y) | Lifetime for those aged 40 to 85y | Population aged 35+ |
| **Type of model** | Markov macro-simulation | Markov | “Spreadsheet” model | “Spreadsheet model” | Markov with dynamic population structure | Markov | Markov cohort |
| **Cost data** | Individual-level administrative data; intervention costs (eg, cost of a law) | Detailed health costs but some intervention costs based on WHO approach | Detailed health costs and some intervention costs (not the food industry agreement) | Intervention costs not specified | Data for health costs, but intervention costs not specified | Data for health costs, but only intervention cost for the collaboration intervention (not tax) | Data for health costs, & simplistic WHO data for intervention cost |
| **Considered health costs from extra life lived** | Yes | Yes | No | No | No | Yes | Not clear |
| **Intervention/s** | Counselling, labelling and mandatory levels | Mandatory reduction of salt content in breads, margarine, and cereals; Community heart health program; In early (2010) paper: also dietary advice and labelling programme | Reduction via assumed food industry agreement; advice for DASH-sodium diets | Legislative (in general) | A population-wide 1g salt per day reduction (by unspecified means) | Collaboration with industry, sodium tax | Not specified population-level reduction |
| **Discount rate (baseline)** | 3% (costs and outcomes) | 3% (costs and outcomes) | 3.5% (costs and outcomes) | 3.5% (costs and outcomes) | 0% (costs and outcomes) | 3% (costs and outcomes) | 3% (only costs) |
| **Main findings** | Generally large QALY gains. Counselling cost-effective, other interventions all cost-saving | Large DALY gain per year (e.g., for mandatory reductions) and cost-saving. The earlier paper also found the labelling programme cost-effective but not the dietary advice. | Large DALY gains. Salt reduction in the population was cost-saving. Dietary advice was sometimes cost-saving, but always cost-effective | Any salt-reduction intervention costing up to £40 million a year would be cost-saving | Large QALY gains. Cost-saving (both salt reduction alone and especially when combined with saturated fat reduction) | Both interventions: large QALY gains and cost saving | Large QALY gains; cost-saving (even if only a modest 1 gm/day reduction were achieved) |
| **Ethnicity/SES considered** | Yes – ethnicity | No | No | No | No | No | Yes – ethnicity |
| **Sensitivity/ scenario analyses** | Yes – extensive | Yes – extensive | Yes – some | Yes – some | Yes – some | Yes – some | Yes – some |
| **Other comments** | Results thought to underestimate the benefits (due to model design). | Many comparisons made with other CVD interventions (2012 paper). Criticised regarding intervention cost details [[23](#_ENREF_23)] | There was a useful comparison with BP treatments. A relatively short time period (10y) was considered. | Assumptions were conservative (eg, benefits over 10y only). For other comment, see also Wang et al [[23](#_ENREF_23)] | Criticised as follows: “Used too many restrictions and assumptions; no particular intervention was specified” [[23](#_ENREF_23)] | Included sensitivity analyses around possible disutility of lower salt diets. Considered tax revenue gained. | Useful comparison with hypertension treatment. African Americans had proportionately greater benefit. |

**^a^ We excluded the cost-saving estimate for a recent UK salt reduction intervention by NICE due to lack of detail [**[**14**](#_ENREF_14)**].**

# Additional methods details

Table B. More detailed explanation of input parameters relating to the interventions effects

| **Intervention** | **Sources and comments** | **Key values and uncertainty (average adult)^a^** |
| --- | --- | --- |
| **Counselling:** Dietary counselling by dietitians to reduce sodium intake (part of current practice). This is specifically for the population with no preceding CVD events. | The full details are in an online report [[24](#_ENREF_24)] but a summary follows. First we estimated the extent of dietary counselling delivered by dietitians in NZ that was considered salt-related (key informant interviews and data from websites and quarterly and annual reports for District Health Boards [DHBs]). This gave a best estimate of 4600 hours of counselling delivered nationally per annum, with a target recipient distribution focused in the 45-64 year age group (64% of the total) but with no relative differences by ethnicity (i.e., DHBs frequently focus counselling on Māori clients whereas the private sector rarely serves Māori). For the effect size on sodium we used the results of the trials included in a 2013 Cochrane systematic review [[25](#_ENREF_25)]. For all the trials in the meta-analysis we calculated hours of counselling provided. | For the per hour impact of counselling: 7.6 mmol/d reduction (with uncertainty based on the initial trials in the Cochrane review. SD=0.8 mmol/d). Normally distributed. Total amount of counselling in NZ: 4600 h/year (SD=920). Gamma distribution. |
| **Endorsement Label Programme:** A programme involving an endorsement label (part of current practice). | The non-governmental organisation “the Heart Foundation” runs an endorsement label programme called the “Tick Programme”. Its estimated impact are in an online report [[26](#_ENREF_26)] and published letter [[27](#_ENREF_27)], but a summary follows. We analysed a 2012 database that covered products for sale by the two main supermarket chains in NZ (the NutriTrack database). This analysis estimated that the Tick Programme was currently changing average sodium intakes for NZ adults by -38 mg/d (i.e., -1.1% of daily intake). Given lack of data on who purchases products with the tick (and diffusion effects within households) we distributed the benefit of consuming “ticked products” over the whole adult population (no relative variation by age-group or ethnicity). | Effect size: 1.7 mmol/d reduction overall (38 mg/d) with SD at +/- 20% (-1.0 to -2.3 mmol/d). Normally distributed. |
| **Mandatory-3G:** Mandatory reduction of sodium in the manufacture of breads, processed meats and sauces (the top 3 categories for sodium intakes in NZ). | The principle of this approach was to follow the recent laws in South Africa [[28](#_ENREF_28)] and various European countries [[29](#_ENREF_29)] mandating limits on sodium levels in certain foods. While detailed in a separate online report [[30](#_ENREF_30)], we summarise here. Based on the relative contributions of sodium to the NZ diet (based on the national nutrition survey data) we estimated the impact of a hypothetical mandatory reduction of sodium in three groups of processed foods: breads, processed meats and sauces (i.e., the top three categories for sodium intake in NZ). A 25% reduction of sodium in each group was assumed to result from setting mandatory upper levels for sodium, giving a reduction in intake of 296 mg/d (12.9 mmol/d). This is a reduction of 8% of current average adult intake. No variation in intake by adult age-group or ethnicity was assumed (given the small overall differences in processed food intakes between Māori and non-Māori [[30](#_ENREF_30)]). | Effect size: 12.9 mmol/d reduction overall with SD at +/- 10% of this. Normally distributed. |
| **Mandatory-All:** Reduction of sodium in all processed foods by 25%. | As above for the Mandatory-3G intervention, except the 25% reduction was applied to all major types of processed foods (i.e., excluding sodium intakes from: fresh fruit and vegetables, fresh fish and meat, and also salt added in cooking and at the table). The estimate obtained was a reduction of sodium intake of 525 mg/d or 22.8 mmol/d (equivalent to 1.4 g/d of salt out of 9.1 g/d salt intake currently or 15% of current adult intake). | Effect size: 22.8 mmol/d reduction overall with SD at +/- 10% of this. Normally distributed. |
| **UK Package:** The mix of media campaign, voluntary food reformulation and food labelling changes | The intervention was that actually used in the 2003-2009 period in the UK but applied on a same per capita basis to NZ. It involved multiple components [[31](#_ENREF_31)], but with major ones being a mass media campaign, activities to encourage industry reformulation of processed foods and changes in food labelling. This overall programme resulted in a 15% reduction in 24-hour urinary sodium over seven years in the adult population (equivalent to a change for the UK population from 9.5 to 8.1 g/d in daily salt intake). We used this reduction in our modelling for the NZ population i.e., a 15% reduction in dietary sodium intake over seven years. In the baseline model we assumed that the benefit would stay in place for the lifetime of the modelled cohort (given the longer-term evidence from countries such as Finland [[32](#_ENREF_32)]). But a scenario analyses included a reversion back to the pre-intervention state (i.e., a linear decline so that after five years there is no remaining benefit of the intervention. | Effect size: 3.2 mmol/d reduction per adult annually over the seven year period (22.7 mmol/d overall) with SD at +/- 10% of this. Normally distributed. |
| **UK Mass Media Campaign:** Just the mass media campaign part of the UK Package | The mass media campaign component of the UK Package was applied on the same per capita basis to NZ. There is evidence that this media campaign increased the proportion of UK adults who made an effort to cut down on salt (i.e., from 34% to 43%) and those trying to reduce salt by checking food labels also increased (i.e., from 29% to 50%) [[31](#_ENREF_31)]. Overall, however, the media campaign has been described as being “not very effective in the long term” [[31](#_ENREF_31)]. Given this information, and the other actions occurring at the time (industry food reformulation) we assumed a relatively modest role for the campaign – at around 30% of the total package effect size (range in scenario analyses of 15% to 45%). This range is very approximate but has been informed by expert opinion [Personal communication with He and Macgregor who have studied the UK campaign [[31](#_ENREF_31)]]. In the baseline model we assumed that the benefit would stay in place for the lifetime of the modelled cohort (i.e., once people got used to less salt in processed foods). But in a scenario analysis we considered a reversion back to the pre-intervention state (i.e., that from the end of the intervention period, there is a linear decline so that after five years there is no remaining benefit of the intervention). | Effect size: 0.97 mmol/d reduction per adult annually over the seven year period (6.8 mmol/d overall) with SD at +/- 30% of this. Normally distributed. |
| **Salt Tax:** An excise tax is applied and increased up to the point where the recommended level of sodium intake is achieved | Fiscal approaches to limiting dietary salt are used in Portugal and Hungary (VAT on salty products and a tax on salty snacks [[29](#_ENREF_29)]) – but no countries specifically have a tax on salt. We modelled a hypothetical intervention in which a law was passed requiring an excise tax on salt that would be applied in increasing amounts annually until a target level of population salt intake of 2300 mg/d (5.9 g salt/d) per adult was achieved (the level recommended for NZ adults [[33](#_ENREF_33)]). We used a price elasticity (PE) for demand of salt from the literature of: -0.1 [[34](#_ENREF_34)] (but varied this in scenario analyses from -0.05 to -0.2). We set the tax levels so that the reduced demand in any one year would never exceed 20%, which approximates a threshold for consumer awareness of salt reduction (i.e., there is evidence that suggests that reductions in bread of up to 20% cannot be perceived and do not influence consumer approval [[35](#_ENREF_35),[36](#_ENREF_36)]). This meant that it took 10 years to reach the 2300 mg/d target. In the baseline model we assumed that the benefit would stay in place for the lifetime of the modelled cohort (given that this is the general pattern with such taxes – albeit the recent discontinuation of the Danish saturated fat tax being a rare exception). Scenario analyses also included the following: (i) The government abandons the Salt Tax at five years after the final tax level is achieved. At this point there is a linear increase in the levels of sodium in processed foods so that after five years there is no remaining benefit of the intervention. (ii) To ensure that the Salt Tax is acceptable to the public, the government also runs the full UK Package of interventions. | Effect size: Variable annual reductions to keep under the maximal level of 20% change in any year. The highest reduction was in the first year at 6.5 mmol/d per adult. |
| **Sinking Lid:** The amount of food-grade salt released onto the NZ market is reduced annually to the point where the recommended level of sodium intake is achieved | In this hypothetical intervention, a law was enacted requiring a stepwise reduction in the amount of food-grade salt released to the market (i.e., as released by NZ’s single salt manufacturer). The reduction continued until the target level of 2300 mg/d per adult was achieved (as per the Salt Tax). In the baseline model we assumed that it would take six years to achieve the target and that the benefit would stay in place for the lifetime of the modelled cohort. Scenario analyses also included the following: (i) A range of longer time frames for the course of the Sinking Lid process were used, at 10 years and 15 years. (ii) It was assumed that the government abandons the Sinking Lid policy at five years after the final target level of food salt released onto the market is achieved (2300 mg/d per average adult). At this point there is a linear increase in the levels of sodium in processed foods so that after five years there is no remaining benefit of the Sinking Lid intervention. (iii) As per a Salt Tax scenario, the UK Package is also run to ensure public acceptability of the Sinking Lid intervention. | Effect size: A reduction in sodium consumption of 9.0 mmol/d per adult each year (until the target is reached). |

^a^ Values given for the average adult. In the modelling we adjusted these values for men and women by ratios of 4013/3544 and 3115/3544 respectively, given the variation in sodium intakes (in mg) according to the nutrition survey data [[37](#_ENREF_37)].

# Additional Results

Table C. Net costs, QALYs and cost-effectiveness incremental compared to “do nothing” by sociodemographic group (expressed per adult in 2011)

| **Intervention / population group** | **Incremental (to “do nothing”) cost per adult in NZ$ (95% UI)** | **QALYs gained per adult (95% UI)** | **ICER (cost in NZ$ per QALY), (95% UI)** |
| --- | --- | --- | --- |
| ***Dietary Counselling*** |  |  |  |
| Age < 65 years | 3.50 | 0.000104 | 33,500 |
| Age 65+ years | 1.70 | 0.000032 | 51,000 |
| Women | 3.40 | 0.000067 | 50,500 |
| Men | 2.60 | 0.000105 | 24,500 |
| Māori | 3.00 (1.80; 4.40) | 0.00014 (0.00007; 0.00023) | 22,900 (13,600; 38,300) |
| Non-Māori | 3.00 (1.80; 4.40) | 0.00008 (0.00004; 0.00013) | 40,200 (23,700; 68,000) |
| ***Endorsement Label Programme*** |  |  |  |
| Age < 65 years | -18.80 | 0.0037 | Dominant |
| Age 65+ years | -3.40 | 0.0027 | Dominant |
| Women | -10.30 | 0.0028 | Dominant |
| Men | -19.90 | 0.0040 | Dominant |
| Māori | -12.00 (-20.00; -6.00) | 0.0044 (0.0031; 0.0058) | Dominant (Dominant to Dominant) |
| Non-Māori | -15.00 (-22.00; -8.00) | 0.0033 (0.0023; 0.0043) | Dominant (Dominant to Dominant) |
| ***Mandatory-3G*** |  |  |  |
| Age < 65 years | -184 | 0.029 | Dominant |
| Age 65+ years | -40.9 | 0.021 | Dominant |
| Women | -114 | 0.022 | Dominant |
| Men | -185 | 0.032 | Dominant |
| Māori | -125 (-174; -85.8) | 0.034 (0.028; 0.042) | Dominant (Dominant to Dominant) |
| Non-Māori | -148 (-193; -109) | 0.026 (0.021; 0.032) | Dominant (Dominant to Dominant) |
| ***Mandatory-All*** |  |  |  |
| Age < 65 years | -327 | 0.051 | Dominant |
| Age 65+ years | -73.5 | 0.038 | Dominant |
| Women | -202 | 0.040 | Dominant |
| Men | -329 | 0.057 | Dominant |
| Māori | -223 (-308; -153) | 0.062 (0.049; 0.074) | Dominant (Dominant to Dominant) |
| Non-Māori | -264 (-348; -195) | 0.046 (0.037; 0.056) | Dominant (Dominant to Dominant) |
| ***UK Package*** |  |  |  |
| Age < 65 years | -244 | 0.042 | Dominant |
| Age 65+ years | -32.8 | 0.023 | Dominant |
| Women | -160 | 0.034 | Dominant |
| Men | -223 | 0.041 | Dominant |
| Māori | -158 (-221; -104) | 0.047 (0.038; 0.057) | Dominant (Dominant to Dominant) |
| Non-Māori | -192 (-250; -142) | 0.036 (0.029; 0.043) | Dominant (Dominant to Dominant) |
| ***UK Mass Media Campaign*** |  |  |  |
| Age < 65 years | -70 | 0.013 | Dominant |
| Age 65+ years | -7.6 | 0.0067 | Dominant |
| Women | -45 | 0.010 | Dominant |
| Men | -64 | 0.012 | Dominant |
| Māori | -44 (-73; -21) | 0.014 (0.008; 0.021) | Dominant (Dominant to Dominant) |
| Non-Māori | -54 (-83; -29) | 0.011 (0.006; 0.015) | Dominant (Dominant to Dominant) |
| ***Salt Tax*** |  |  |  |
| Age < 65 years | -558 | 0.097 | Dominant |
| Age 65+ years | -74 | 0.048 | Dominant |
| Women | -368 | 0.077 | Dominant |
| Men | -507 | 0.094 | Dominant |
| Māori | -357 (-499; -240) | 0.110 (0.090; 0.130) | Dominant (Dominant to Dominant) |
| Non-Māori | -438 (-578; -322) | 0.080 (0.066; 0.100) | Dominant (Dominant to Dominant) |
| ***Sinking Lid*** |  |  |  |
| Age < 65 years | -613 | 0.100 | Dominant |
| Age 65+ years | -95 | 0.058 | Dominant |
| Women | -405 | 0.083 | Dominant |
| Men | -564 | 0.102 | Dominant |
| Māori | -405 (-558; -273) | 0.120 (0.094; 0.140) | Dominant (Dominant to Dominant) |
| Non-Māori | -488 (-638; -363) | 0.090 (0.071; 0.110) | Dominant (Dominant to Dominant) |

# Scenario Analyses – Extra Methods and Results

For some of the scenario analyses considered, the Counselling intervention was not always cost-effective (e.g., at the 6% discount rate) (S4 Table and S5 Table). This was also the case when we used the results of another meta-analysis for the relationship between sodium intake and blood pressure [[38](#_ENREF_38)] (this pushed up the ICER to NZ$ 64,800 per QALY gained). Also, the Counselling intervention became cost-saving if it was shifted from individual-level counselling to group counselling.

A possible limitation with the use of HealthTracker data was that we may have over-estimated the case-fatality rates (CFRs) for CHD and stroke (given that the look-back period would have missed out on incident cases earlier than the 10-year period used). Using DisMod we estimated that this over-estimate in the 60+ years age group might conceivably be as follows for CHD, ischaemic stroke and haemorrhagic stroke respectively in non-Māori women (0%, 35%, 25%); in non-Māori men (20%, 40%, 35%); in Māori women (40%, 40%, 30%); and in Māori men (50%, 60%, 65%). Hence we conducted scenario analyses using these values to produce lower CFRs (S4 Table). This resulted in somewhat lower QALY gains by an average of 10% and lower net health system costs (by an average of 27%, probably). So the pattern of all (but one) interventions being cost saving did not change and the ICER for dietary counselling only increased marginally from $35,000 to $37,000 per QALY gained.

For all the interventions there were large increases in QALYs gained when using a 0% discount rate. When using a 6% discount rate the cost-saving interventions achieved lower savings (e.g., reduced by around a quarter for the Sinking Lid).

Variation in the price elasticity of the demand for salt by food manufacturers (from -0.05 to -0.2) did change the results notably, but all scenarios were still cost-saving (S4 Table). Various interventions were still found to be worthwhile even if the benefits declined over time (e.g., the UK Package) and if the Salt Tax and the Sinking Lid interventions were abandoned by the government five years after implementation.

To provide additional context for these results, we also ran a scenario where we lowered population sodium intakes to the level of the theoretical minimum risk exposure distribution (TMRED) used in the Global Burden of Disease Study 2010 [[39](#_ENREF_39)] (i.e., 1000 mg sodium per day). This resulted in a relatively large impact in discounted QALYs gained (i.e., 0.24 per an individual adult) or 2.6 times the gain in the Sinking Lid intervention. To provide further context, we also considered the results from a hypothetical intervention (e.g., a vaccine) that eradicated CVD entirely in the adult population. This resulted in 1.28 QALYs gained per individual adult (i.e., around 15 months of extra life in full health), or eight times the gain in the Sinking Lid intervention.

Table D. Scenario analysis relating to the sodium reduction interventions (cost, health gain and cost-effectiveness per individual New Zealand adult)

| **Additional scenario analyses – intervention parameters** | **Incre-mental cost per adult (NZ$)** | **QALY gain per adult** | **ICER (cost NZ$ per QALY)** |
| --- | --- | --- | --- |
| ***Dietary Counselling****(current practice)* | | | |
| Best Scenario: Full effect for each person counselled lasts for 3 years then declines linearly to zero at 3 years after that, 3% discount rate | 3.01 | 0.000085 | 35,200 |
| Scenario 1A: Full effect for 3 years then zero effect | 3.19 | 0.000060 | 52,900 |
| Scenario 1B: Full effect for 3 years then declines linearly to zero at 5 years after that | 2.90 | 0.000101 | 28,900 |
| Scenario 1C: Lower cost from only having group counselling (n=10 per group) | -0.11 | 0.000085 | Dominant |
| Lower case-fatality rates for CHD and stroke (as per *Methods*) | 2.89 | 0.000077 | 37,400 |
| 0% discount rate (otherwise same as ‘best’) | 4.85 | 0.000199 | 24,300 |
| 6% discount rate (otherwise same as ‘best’) | 2.17 | 0.000042 | 51,500 |
| ***Endorsement Label Programme*** *(current practice)* | | | |
| Best Scenario: Assumes the whole cost of the programme is for sodium reduction, 3% discount rate | -14.9 | 0.0034 | Dominant |
| Scenario 2A: Half the cost of the Endorsement Label Programme on the assumption that the health goals of this Programme were equally shared between sodium reduction and saturated fat reduction (since it achieves both [[26](#_ENREF_26)]). | -16.9 | 0.0034 | Dominant |
| Scenario 2B: Varying standard deviation (SD) of sodium reduction (10% of the mean) | -15.0 (-20.9; -9.8) | 0.0034 (0.0028; 0.0042) | Dominant (Dominant to Dominant) |
| Scenario 2C: Varying SD of sodium reduction (40% of the mean) | -14.8 (-27.4; -4.3) | 0.0034 (0.0016; 0.0054) | Dominant (Dominant to Dominant) |
| Lower case-fatality rates for CHD and stroke (as per *Methods*) | -20.3 | 0.0031 | Dominant |
| 0% discount rate (otherwise same as ‘best’) | -13.2 | 0.0078 | Dominant |
| 6% discount rate (otherwise same as ‘best’) | -12.2 | 0.0018 | Dominant |
| ***Mandatory-3G*** | | | |
| Best Scenario: Law stays in place permanently, 3% discount rate | -147 | 0.027 | Dominant |
| Scenario 3A: Assume that the benefit of the law persists for 20 years and then expires (sunset clause) | -121 | 0.019 | Dominant |
| Scenario 3B: Assuming half of the annual Australian enforcement costs | -143 | 0.027 | Dominant |
| Lower case-fatality rates for CHD and stroke (as per *Methods*) | -190 | 0.024 | Dominant |
| 0% discount rate (otherwise same as ‘best’) | -151 | 0.062 | Dominant |
| 6% discount rate (otherwise same as ‘best’) | -117 | 0.014 | Dominant |
| ***Mandatory-All*** | | | |
| Best Scenario: Law stays in place permanently, 3% discount rate | -262 | 0.048 | Dominant |
| Scenario 4A: Assume that the benefit of the law persists for 20 years and then expires (sunset clause) | -215 | 0.033 | Dominant |
| Scenario 4B: Assuming half of the annual Australian enforcement costs | -259 | 0.048 | Dominant |
| Lower case-fatality rates for CHD and stroke (as per *Methods*) | -338 | 0.043 | Dominant |
| 0% discount rate (otherwise same as ‘best’) | -269 | 0.110 | Dominant |
| 6% discount rate (otherwise same as ‘best’) | -209 | 0.024 | Dominant |
| ***UK Package*** |  |  |  |
| Best Scenario: Intervention cost and effect as per the UK experience, 3% discount rate | -190 | 0.037 | Dominant |
| Intervention cost up 50% (otherwise same as ‘best’) | -188 | 0.037 | Dominant |
| Rather than the effect persisting into the distant future (as in ‘best’), there is reversion back to the pre-intervention sodium intake levels (from the end of the intervention period, there is a linear decline so that after five years there is no remaining benefit of the intervention) | -67.3 | 0.011 | Dominant |
| Lower case-fatality rates for CHD and stroke (as per *Methods*) | -249 | 0.033 | Dominant |
| 0% discount rate (otherwise same as ‘best’) | -205 | 0.090 | Dominant |
| 6% discount rate (otherwise same as ‘best’) | -141 | 0.018 | Dominant |
| ***UK Mass Media Campaign*** |  |  |  |
| Best Scenario: Intervention cost and effect as per the UK experience, benefit equals 30% of the UK package, 3% discount rate | -54.1 | 0.011 | Dominant |
| Benefit equals 15% of the whole UK package (otherwise same as ‘best’) | -25.0 | 0.0055 | Dominant |
| Benefit equals 45% of the whole UK package (otherwise same as ‘best’) | -83.3 | 0.017 | Dominant |
| Intervention cost up 50% (otherwise same as ‘best’) | -52.1 | 0.011 | Dominant |
| There is reversion back to the pre-intervention state sodium intake levels post-campaign | -17.5 | 0.0032 | Dominant |
| Lower case-fatality rates for CHD and stroke (as per *Methods*) | -71.5 | 0.0099 | Dominant |
| 0% discount rate (otherwise same as ‘best’) | -58.6 | 0.027 | Dominant |
| 6% discount rate (otherwise same as ‘best’) | -39.4 | 0.0052 | Dominant |
| ***Salt Tax*** |  |  |  |
| Best Scenario: Time frame is 10 years, PE = -0.1, 3% discount rate | -435 | 0.085 | Dominant |
| Price elasticity is lower: -0.05 (otherwise same as ‘best’) | -236 | 0.046 | Dominant |
| Price elasticity is higher: -0.2 (otherwise same as ‘best’) | -503 | 0.095 | Dominant |
| The government abandons the salt tax at five years after the final tax level is achieved and sodium intakes return to pre-intervention levels | -291 | 0.045 | Dominant |
| The UK Package is also run to ensure public acceptability of the tax intervention | -430 | 0.085 | Dominant |
| Lower case-fatality rates for CHD and stroke (as per *Methods*) | -570 | 0.076 | Dominant |
| 0% discount rate (otherwise same as ‘best’) | -470 | 0.210 | Dominant |
| 6% discount rate (otherwise same as ‘best’) | -319 | 0.040 | Dominant |
| ***Sinking Lid*** |  |  |  |
| Best Scenario: Phase-in time frame is six years, 3% discount rate | -481 | 0.092 | Dominant |
| Time frame is 10 years (otherwise same as ‘best’) | -425 | 0.083 | Dominant |
| Time frame is 15 years (otherwise same as ‘best’) | -362 | 0.074 | Dominant |
| The government abandons the sinking lid policy at five years after the final target level of food salt released onto the market is achieved | -268 | 0.040 | Dominant |
| The UK Package is also run to ensure public acceptability of the sinking lid intervention | -476 | 0.092 | Dominant |
| Lower case-fatality rates for CHD and stroke (as per *Methods*) | -626 | 0.083 | Dominant |
| 0% discount rate (otherwise same as ‘best’) | -513 | 0.220 | Dominant |
| 6% discount rate (otherwise same as ‘best’) | -361 | 0.044 | Dominant |
| ***Hypothetical comparison interventions to provide context*** |  |  |  |
| Sodium intakes are reduced to the theoretical minimum risk exposure distribution (TMRED) of 1000 mg per day | - | 0.24 | - |
| All CVD eradicated | - | 1.28 | - |
| As above for CVD eradication but discount rate = 0% | - | 3.16 | - |

Table E. Scenario analysis relating to changing a key epidemiological parameter – the relationship between sodium intake reduction and systolic blood pressure reduction (alternative meta-analysis by He & Macgregor 2013 [[38](#_ENREF_38)])

| **Intervention scenario** | **Health system cost (NZ$; millions) for remainder of the cohort’s life (95%UI)** | **QALYs for remainder of the cohort’s life (95%UI)** | **ICER [cost in NZ$ per QALY] (95%UI)** |
| --- | --- | --- | --- |
| “Do nothing” comparator | 162,000 | 33,200,000 | Not applicable |
|  | (145,000 to 181,000) | (33,000,000 to 33,400,000) |  |
| – Alternative meta-analysis | 161,000 | 33,400,000 | Not applicable |
|  | (144,000 to 179,000) | (33,200,000 to 33,600,000) |  |
| ***Incremental to “Do Nothing”*** |  |  |  |
| Counselling intervention | 6.9 | 200 | 36,900 |
|  | (4.2 to 10.2) | (100 to 330) | (22,400 to 62,500) |
| – Alternative meta-analysis | 7.7 | 130 | 64,800 |
|  | (4.9 to 11.2) | (70 to 210) | (40,500 to 109,200) |
| Endorsement Label Programme | -34 | 7900 | Dominant |
|  | (-52 to -19) | (5500 to 10,400) |  |
| – Alternative meta-analysis | -18 | 5100 | Dominant |
|  | (-30 to -9) | (3600 to 6700) |  |
| Mandatory-3G intervention | -340 | 61,700 | Dominant |
|  | (-440 to -240) | (49,700 to 74,900) |  |
| – Alternative meta-analysis | -210 | 40,100 | Dominant |
|  | (-280 to -150) | (31,900 to 48,000) |  |
| Mandatory-All intervention | -600 | 110,400 | Dominant |
|  | (-800 to -440) | (87,500 to 135,000) |  |
| – Alternative meta-analysis | -380 | 71,200 | Dominant |
|  | (-490 to -300) | (56,500 to 85,900) |  |
| UK Package | -440 | 85,100 | Dominant |
|  | (-570 to -320) | (67,600 to 102,000) |  |
| – Alternative meta-analysis | -270 | 55,200 | Dominant |
|  | (-360 to -200) | (44,900 to 66,900) |  |
| Media campaign component of UK Package | -120 | 25,200 | Dominant |
|  | (-200 to -62) | (14,200 to 36,700) |  |
| – Alternative meta-analysis | -75 | 16,600 | Dominant |
|  | (-120 to -37) | (9600 to 24,300) |  |
| Salt tax | -1000 | 195,000 | Dominant |
|  | (-1320 to -740) | (159,000 to 237,000) |  |
| – Alternative meta-analysis | -620 | 125,800 | Dominant |
|  | (-820 to -450) | (101,000 to 154,000) |  |
| Sinking lid | -1110 | 211,000 | Dominant |
|  | (-1460 to -830) | (170,000 to 255,000) |  |
| – Alternative meta-analysis | -690 | 135,000 | Dominant |
|  | (-910 to -510) | (108,000 to 167,000) |  |

# Uncertainty analysis – tornado plots

Fig. A: Tornado plots for univariate sensitivity analyses using the 2.5^th^ and 97.5^th^ percentile of input parameters on incremental cost and QALYs gained per individual NZ adult (Counselling and Mandatory-All) and ICER (Counselling only)^a^

1. Incremental cost, Counselling compared to Do Nothing

**^a^** Explanatory notes for terms in the tornado plots: Sodium reduction: Reduction in sodium intake (mmol/d) as a result of the intervention (e.g., Counselling intervention in this case); Effect size of BP change for stroke risk: The change in stroke risk for a one percent change in sBP; Effect size of BP change for CHD risk: The change in CHD risk for a one percent change in sBP; Disease cost for CHD: CHD health system costs; Disease cost for stroke: Stroke health system costs; Other health system cost: Non-CVD health system costs; Health system cost for intervention: Direct intervention cost.

1. Incremental QALYs, Counselling compared to Do Nothing
2. ICERs, Counselling compared to Do Nothing
3. Incremental cost, Mandatory-All compared to Do Nothing
4. Incremental QALYs, Mandatory-All compared to Do Nothing

# Further details on the limitations of this modelling work

**Model structure.** The model structure involved some simplification in that other health conditions associated with high sodium intakes were not considered e.g., stomach cancer [[40](#_ENREF_40)], renal disease [[41](#_ENREF_41)], and possibly even obesity given the association between sodium intake and that of sugar-sweetened beverages [[42](#_ENREF_42)]. Furthermore, we did not include any disutility for the state of having high blood pressure or diagnosed hypertension (which may involve disutilities associated with taking anti-hypertensive medications). The modelling of just the 35+ plus age-group meant that for most of the interventions there was no health benefit assigned to younger populations that would age into the 35+ age group over time. That is the health benefit to these population groups from lower sodium intakes in slowing BP rise with age from these interventions was not counted (resulting in likely under-estimation of the total health benefit).

**Various input parameters.** There are limitations with the cost data from HealthTracker and so some scaling was required (as detailed in the *Methods Section*). There is ongoing work to address these limitations – and therefore future improvements in costing quality are likely.

There was substantial uncertainty around the effect of media campaign component of the UK Package, though we included a range (15% to 45%) in the scenario analyses. Similarly, there is uncertainty around the price elasticity of demand associated with any increased price of salt from a tax on salt (we found few estimates in the published literature). In particular, there is uncertainty around the effectiveness of a potential Sinking Lid intervention (e.g., it is somewhat unclear how effectively the amount of sodium in imported food be constrained as part of a country-level Sinking Lid programme).

The Counselling intervention data were partly based on expert opinion and region-specific administrative data, rather than comprehensive national survey data (other limitations are expanded on an online report [[24](#_ENREF_24)]). Furthermore, the trial data may overestimate the benefits of counselling interventions in the community given the evidence from a systematic review that compliance with low sodium diets “was generally low when only counselling service was given without food supplies” [[43](#_ENREF_43)]. Similarly, the Endorsement Label Programme data and related analysis had various limitations [[26](#_ENREF_26)], including around the nature of the counterfactual used (e.g., if no such Programme existed then it is possible that increased marketing of low sodium foods might occur). There was also an assumption that data on available products in the sampled supermarkets was reflective of sales (though this may not be too important given UK data [[44](#_ENREF_44)]). Our analysis also did not consider how food manufacturers might lower sodium in a range of products even if only some products ended up getting Tick certified (as found in an Australian study [[45](#_ENREF_45)]), resulting in a likely underestimation of the Endorsement Label Programme’s impact.

**Unknowns in public and industry responses.** For all the interventions there was also no consideration of the public response to consuming home-made or processed foods with lower sodium e.g., would some people tend to subsequently add more salt (or salty sauces) at the table to compensate for less salty food? Furthermore, the food industry might respond in unpredictable ways that might counter some of the health gains e.g., by increasing sugar and saturated fat levels in the foods with reduced sodium (to potentially enhance perceived palatability). On the other hand the food industry could further increase use of potassium chloride (KCl) in processed foods to retain the salty taste, with this extra potassium potentially being beneficial for health [[46](#_ENREF_46)] (and this meta-analysis [[47](#_ENREF_47)]). This approach has been purposefully used in Finland since the 1980s and is one of the key ways to lower sodium while retaining a salty taste [[48](#_ENREF_48),[49](#_ENREF_49)].

**Only a health sector perspective.** As we took a health system perspective, some costs and benefits were out-of-scope. Such costs include: (i) the cost to industry of food reformulation and new nutrition labelling on food packaging in response to new laws relating to sodium supply; (ii) and potentially any temporarily reduced eating pleasure for people associated with consuming lower sodium food (e.g., if they actually noticed and before their taste perception generally adapted to these foods in subsequent months). On the other hand, less sodium in processed meats could potentially improve value-for-money for consumers (given how sodium can increase water retention in such meats which increases their weight for potentially no added dietary or culinary “value” to consumers). Also if processed foods are less salty, then sodium-induced thirst may also decline with resultant convenience and other benefits. For example, people eating snacks that are less salty might be less likely to over-consume soft drinks or alcohol (with the associated surplus energy and financial costs involved). Indeed, once people are on lower sodium diets they may actually prefer them, according to work that that has measured the hedonic value of dietary sodium [[50](#_ENREF_50)].

**Limits around generalisability**

The results in this study probably have a reasonable level of applicability to other countries – given that high sodium intakes are a risk to health in virtually every country and most governments have the potential powers to legislate around sodium in the food supply. But for some of the interventions there will of course be specific considerations around feasibility and effectiveness. For example, a Sinking Lid might be relatively simple in a country like New Zealand (with a single producer of food-grade salt), but more complex in nations with multiple producers and importers. For the UK Package, the effect may depend on the responsiveness of the food industry in a country to its government calls to lower sodium levels in processed foods. Similarly, the impact of a mass media campaign (as used in the UK) will depend on public knowledge and receptivity to such messages and other informational materials in the food environment such as the type of nutrition labelling on processed foods. Furthermore, in some countries it might be more feasible and acceptable to have group counselling than the individual-level counselling that was modelled here.

**References**

1. Joffres MR, Campbell NR, Manns B, Tu K (2007) Estimate of the benefits of a population-based reduction in dietary sodium additives on hypertension and its related health care costs in Canada. Can J Cardiol 23: 437-443.

2. Asaria P, Chisholm D, Mathers C, Ezzati M, Beaglehole R (2007) Chronic disease prevention: health effects and financial costs of strategies to reduce salt intake and control tobacco use. Lancet 370: 2044-2053.

3. Hendriksen MA, Hoogenveen RT, Hoekstra J, Geleijnse JM, Boshuizen HC, et al. (2014) Potential effect of salt reduction in processed foods on health. Am J Clin Nutr 99: 446-453.

4. Dall TM, Fulgoni VL, 3rd, Zhang Y, Reimers KJ, Packard PT, et al. (2009) Potential health benefits and medical cost savings from calorie, sodium, and saturated fat reductions in the American diet. Am J Health Promot 23: 412-422.

5. Palar K, Sturm R (2009) Potential societal savings from reduced sodium consumption in the U.S. adult population. Am J Health Promot 24: 49-57.

6. Martikainen JA, Soini EJ, Laaksonen DE, Niskanen L (2011) Health economic consequences of reducing salt intake and replacing saturated fat with polyunsaturated fat in the adult Finnish population: estimates based on the FINRISK and FINDIET studies. Eur J Clin Nutr 65: 1148-1155.

7. Gase LN, Kuo T, Dunet D, Schmidt SM, Simon PA, et al. (2011) Estimating the potential health impact and costs of implementing a local policy for food procurement to reduce the consumption of sodium in the county of Los Angeles. Am J Public Health 101: 1501-1507.

8. Rubinstein A, Colantonio L, Bardach A, Caporale J, Marti SG, et al. (2010) Estimation of the burden of cardiovascular disease attributable to modifiable risk factors and cost-effectiveness analysis of preventative interventions to reduce this burden in Argentina. BMC Public Health 10: 627.

9. Ferrante D, Konfino J, Mejia R, Coxson P, Moran A, et al. (2012) The cost-utility ratio of reducing salt intake and its impact on the incidence of cardiovascular disease in Argentina [In Spanish]. Rev Panam Salud Publica 32: 274-280.

10. Cobiac LJ, Vos T, Veerman JL (2010) Cost-effectiveness of interventions to reduce dietary salt intake. Heart 96: 1920-1925.

11. Cobiac LJ, Magnus A, Lim S, Barendregt JJ, Carter R, et al. (2012) Which interventions offer best value for money in primary prevention of cardiovascular disease? PLoS One 7: e41842.

12. Dodhia H, Phillips K, Zannou MI, Airoldi M, Bevan G (2012) Modelling the impact on avoidable cardiovascular disease burden and costs of interventions to lower SBP in the England population. J Hypertens 30: 217-226.

13. Barton P, Andronis L, Briggs A, McPherson K, Capewell S (2011) Effectiveness and cost effectiveness of cardiovascular disease prevention in whole populations: modelling study. BMJ 343: d4044.

14. NICE (National Institute for Health and Care Excellence) (2010) Prevention of cardiovascular disease. NICE public health guidance 25. London: NICE.

15. Selmer RM, Kristiansen IS, Haglerod A, Graff-Iversen S, Larsen HK, et al. (2000) Cost and health consequences of reducing the population intake of salt. J Epidemiol Community Health 54: 697-702.

16. Bibbins-Domingo K, Chertow GM, Coxson PG, Moran A, Lightwood JM, et al. (2010) Projected effect of dietary salt reductions on future cardiovascular disease. N Engl J Med 362: 590-599.

17. Carter MC, Burley VJ, Nykjaer C, Cade JE (2013) Adherence to a smartphone application for weight loss compared to website and paper diary: pilot randomized controlled trial. J Med Internet Res 15: e32.

18. Mason H, Shoaibi A, Ghandour R, O'Flaherty M, Capewell S, et al. (2014) A cost effectiveness analysis of salt reduction policies to reduce coronary heart disease in four Eastern Mediterranean countries. PLoS One 9: e84445.

19. Ha DA, Chisholm D (2011) Cost-effectiveness analysis of interventions to prevent cardiovascular disease in Vietnam. Health Policy Plan 26: 210-222.

20. Murray CJ, Lauer JA, Hutubessy RC, Niessen L, Tomijima N, et al. (2003) Effectiveness and costs of interventions to lower systolic blood pressure and cholesterol: a global and regional analysis on reduction of cardiovascular-disease risk. Lancet 361: 717-725.

21. Salomon JA, Carvalho N, Gutierrez-Delgado C, Orozco R, Mancuso A, et al. (2012) Intervention strategies to reduce the burden of non-communicable diseases in Mexico: cost effectiveness analysis. BMJ 344: e355.

22. Wang G, Labarthe D (2011) The cost-effectiveness of interventions designed to reduce sodium intake. J Hypertens 29: 1693-1699.

23. Wang G, Bowman BA (2013) Recent economic evaluations of interventions to prevent cardiovascular disease by reducing sodium intake. Curr Atheroscler Rep 15: 349.

24. Wilson N (2014) Technical Report: Estimating the modelling parameters around dietary counselling for preventing cardiovascular disease in New Zealand. Wellington: University of Otago, Wellington. <http://www.otago.ac.nz/wellington/otago071960.pdf>.

25. Rees K, Dyakova M, Ward K, Thorogood M, Brunner E (2013) Dietary advice for reducing cardiovascular risk. Cochrane Database Syst Rev 3: CD002128.

26. Wilson N, Nghiem N (2014) Background Report for BODE3 Modelling on Estimating the Impact of the Tick Programme in New Zealand (a Heart Health Food Endorsement Programme). Wellington: University of Otago, Wellington. <http://www.otago.ac.nz/wellington/otago071961.pdf>.

27. Wilson N, Nghiem N, Eyles H, Ni Mhurchu C, Cobiac LJ, et al. (2014) Possible impact of the Tick Programme in New Zealand on selected nutrient intakes: Tentative estimates and methodological complexities. N Z Med J 127(1399): 85-88.

28. Hofman K, Tollman S (2013) Population health in South Africa: a view from the salt mines. Lancet Global Health 1: e66-67.

29. European Commission Survey on members states implementation of the EU salt reduction framework: Directorate-General Health and Consumers 2012. <http://ec.europa.eu/health/nutrition_physical_activity/docs/salt_report1_en.pdf>

30. Wilson N (2014) Background Technical Details on Mandatory Interventions Used in Sodium Reduction Modelling for Cardiovascular Disease Prevention. Wellington: Department of Public Health, University of Otago. <http://www.otago.ac.nz/wellington/otago072196.pdf>

31. He FJ, Brinsden HC, Macgregor GA (2013) Salt reduction in the United Kingdom: a successful experiment in public health. J Hum Hypertens [E-publication 31 October].

32. Pietinen P, Mannisto S, Valsta LM, Sarlio-Lahteenkorva S (2010) Nutrition policy in Finland. Public Health Nutr 13: 901-906.

33. NHMRC/MoH (2006) Nutrient Reference Values for Australia and New Zealand. Canberra, ACT: National Health and Medical Research Council (NHMRC); New Zealand Ministry of Health (MoH). <http://www.nhmrc.gov.au;> <http://www.moh.govt.nz/publications>

34. Landsburg S (2010) Price Theory and Applications (with Economic Applications): Joe Sabatino.

35. Girgis S, Neal B, Prescott J, Prendergast J, Dumbrell S, et al. (2003) A one-quarter reduction in the salt content of bread can be made without detection. European Journal of Clinical Nutrition 57: 616-620.

36. Rodgers A, Neal B (1999) Less salt does not necessarily mean less taste. Lancet 353: 1332.

37. McLean R, Williams S, Mann J, Parnell W (2011) How much salt are we eating? Estimates of New Zealand population sodium from the 2008/2009 Adult Nutrition Survey [Presentation on 2 December 2011]. Joint Annual Scientific Meeting of the Australian and New Zealand Nutrition Societies. Queenstown, New Zealand (29 November - 2 December).

38. He FJ, Li J, Macgregor GA (2013) Effect of longer-term modest salt reduction on blood pressure. Cochrane Database Syst Rev 4: CD004937.

39. Lim SS, Vos T, Flaxman AD, Danaei G, Shibuya K, et al. (2012) A comparative risk assessment of burden of disease and injury attributable to 67 risk factors and risk factor clusters in 21 regions, 1990-2010: a systematic analysis for the Global Burden of Disease Study 2010. Lancet 380: 2224-2260.

40. D'Elia L, Rossi G, Ippolito R, Cappuccio FP, Strazzullo P (2012) Habitual salt intake and risk of gastric cancer: a meta-analysis of prospective studies. Clin Nutr 31: 489-498.

41. Smyth A, O'Donnell MJ, Yusuf S, Clase CM, Teo KK, et al. (2014) Sodium Intake and Renal Outcomes: A Systematic Review. Am J Hypertens 27: 1277-84.

42. He FJ, Marrero NM, MacGregor GA (2008) Salt intake is related to soft drink consumption in children and adolescents: a link to obesity? Hypertension 51: 629-634.

43. Kwan MW, Wong MC, Wang HH, Liu KQ, Lee CL, et al. (2013) Compliance with the Dietary Approaches to Stop Hypertension (DASH) Diet: A Systematic Review. PLoS One 8: e78412.

44. Eyles H, Webster J, Jebb S, Capelin C, Neal B, et al. (2013) Impact of the UK voluntary sodium reduction targets on the sodium content of processed foods from 2006 to 2011: Analysis of household consumer panel data. Prev Med 57: 555-560.

45. Williams P, McMahon A, Boustead R (2003) A case study of sodium reduction in breakfast cereals and the impact of the Pick the Tick food information program in Australia. Health Promot Int 18: 51-56.

46. Aburto NJ, Hanson S, Gutierrez H, Hooper L, Elliott P, et al. (2013) Effect of increased potassium intake on cardiovascular risk factors and disease: systematic review and meta-analyses. BMJ 346: f1378.

47. Peng YG, Li W, Wen XX, Li Y, Hu JH, et al. (2014) Effects of salt substitutes on blood pressure: a meta-analysis of randomized controlled trials. Am J Clin Nutr 100: 1448-1454.

48. Dotsch M, Busch J, Batenburg M, Liem G, Tareilus E, et al. (2009) Strategies to reduce sodium consumption: a food industry perspective. Crit Rev Food Sci Nutr 49: 841-851.

49. Batenburg M, van der Velden R (2011) Saltiness enhancement by savory aroma compounds. J Food Sci 76: S280-288.

50. Blais CA, Pangborn RM, Borhani NO, Ferrell MF, Prineas RJ, et al. (1986) Effect of dietary sodium restriction on taste responses to sodium chloride: a longitudinal study. Am J Clin Nutr 44: 232-243.
