# Supplementary material for: Health and Economic Impacts of Eight Different Dietary Salt Reduction Interventions
Source: PLoS One. 2015 Apr 24;10(4):e0123915. doi: 10.1371/journal.pone.0123915 (PMC4409110; doi:10.1371/journal.pone.0123915)
Supplement: S2 File — (DOCX) [file pone.0123915.s002.docx]

**Supporting Information – Costs (S2)**

**(For Nghiem et al – “Health and Economic Impacts of Eight Different Dietary Salt Reduction Interventions”)**

| **S6 Table. Excess CVD treatment costs (NZ$/adult/year) for the first year in 2011 value by**  **age and sex** | | | | | |
| --- | --- | --- | --- | --- | --- |
|  |  |  |  |  |  |
| **Disease** | **Coronary heart disease (CHD)** | | **Stroke** | |  |
| **Age group** | **Women** | **Men** | **Women** | **Men** |  |
| 35-39 | 18,412 | 17,515 | 23,669 | 24,195 |  |
| 40-44 | 18,545 | 17,314 | 23,869 | 23,894 |  |
| 45-49 | 18,388 | 17,064 | 23,635 | 23,520 |  |
| 50-54 | 17,086 | 18,614 | 21,796 | 18,573 |  |
| 55-59 | 16,745 | 18,161 | 21,285 | 17,895 |  |
| 60-64 | 16,258 | 17,569 | 20,553 | 17,006 |  |
| 65-69 | 16,448 | 19,733 | 15,565 | 15,866 |  |
| 70-74 | 15,777 | 18,784 | 14,558 | 14,442 |  |
| 75-79 | 15,138 | 18,053 | 13,600 | 13,346 |  |
| 80-84 | 9,921 | 13,303 | 10,560 | 11,594 |  |
| 85-89 | 9,636 | 12,955 | 10,133 | 11,071 |  |
| 90+ | 8,065 | 9,467 | 8,537 | 10,117 |  |
|  |  |  |  |  |  |
| **S7 Table. Excess CVD treatment costs (NZ$/adult/year) for second and subsequent years**  **in 2011 value by age and sex** | | | | | |
|  |  |  |  |  |  |
| **Disease** | **CHD** | | **Stroke** | |  |
| **Age group** | **Women** | **Men** | **Women** | **Men** |  |
| 35-39 | 6,281 | 5,179 | 7,455 | 6,495 |  |
| 40-44 | 6,414 | 4,979 | 7,655 | 6,195 |  |
| 45-49 | 6,258 | 4,729 | 7,420 | 5,820 |  |
| 50-54 | 6,224 | 4,625 | 7,234 | 8,654 |  |
| 55-59 | 5,882 | 4,173 | 6,722 | 7,975 |  |
| 60-64 | 5,395 | 3,581 | 5,991 | 7,087 |  |
| 65-69 | 5,217 | 4,390 | 6,930 | 7,180 |  |
| 70-74 | 4,546 | 3,441 | 5,923 | 5,757 |  |
| 75-79 | 3,908 | 2,710 | 4,965 | 4,660 |  |
| 80-84 | 3,374 | 3,213 | 3,742 | 4,228 |  |
| 85-89 | 3,089 | 2,865 | 3,315 | 3,705 |  |
| 90+ | 2,426 | 3,047 | 2,516 | 2,968 |  |
|  |  |  |  |  |  |
|  |  |  |  |  |  |

| **S8 Table. Average citizen health costs excluding CVD costs (NZ$/adult/year) in 2011**  **dollars by age and sex** | | | | | |
| --- | --- | --- | --- | --- | --- |
|  |  |  |  |  |  |
| **Age group** | **Women** | **Men** |  |  |  |
| 35-39 | 863 | 1,368 |  |  |  |
| 40-44 | 1,021 | 1,214 |  |  |  |
| 45-49 | 1,197 | 1,330 |  |  |  |
| 50-54 | 1,483 | 1,552 |  |  |  |
| 55-59 | 1,835 | 1,828 |  |  |  |
| 60-64 | 2,381 | 2,251 |  |  |  |
| 65-69 | 3,167 | 2,955 |  |  |  |
| 70-74 | 4,204 | 3,535 |  |  |  |
| 75-79 | 5,049 | 4,027 |  |  |  |
| 80-84 | 5,598 | 4,679 |  |  |  |
| 85-89 | 6,411 | 5,116 |  |  |  |
| 90+ | 6,552 | 5,050 |  |  |  |
